# Research progress of extracellular vesicles and exosomes derived from mesenchymal stem cells in the treatment of oxidative stress-related diseases

**DOI:** 10.3389/fimmu.2023.1238789

**Published:** 2023-08-14

**Authors:** Wenwen Zhang, Tingyu Wang, Yuanye Xue, Bingbing Zhan, Zengjie Lai, Wenjie Huang, Xinsheng Peng, Yanfang Zhou

**Affiliations:** ^1^ The First Dongguan Affiliated Hospital of Guangdong Medical University, Dongguan, Guangdong, China; ^2^ Department of Pathophysiology, Guangdong Medical University, Dongguan, Guangdong, China; ^3^ School of Pharmaceutical Sciences, Guangdong Medical University, Dongguan, China; ^4^ The Second Clinical Medical College of Guangdong Medical University, Dongguan, China; ^5^ School of Medical Technology, Guangdong Medical University, Dongguan, China; ^6^ Biomedical Innovation Center, Guangdong Medical University, Dongguan, China; ^7^ Institute of Marine Medicine, Guangdong Medical University, Zhanjiang, China

**Keywords:** mesenchymal stem cells, extracellular vesicles, exosomes, oxidative stress, inflammation, cell proliferation

## Abstract

There is growing evidence that mesenchymal stem cell-derived extracellular vesicles and exosomes can significantly improve the curative effect of oxidative stress-related diseases. Mesenchymal stem cell extracellular vesicles and exosomes (MSC-EVs and MSC-Exos) are rich in bioactive molecules and have many biological regulatory functions. In this review, we describe how MSC-EVs and MSC-Exos reduce the related markers of oxidative stress and inflammation in various systemic diseases, and the molecular mechanism of MSC-EVs and MSC-Exos in treating apoptosis and vascular injury induced by oxidative stress. The results of a large number of experimental studies have shown that both local and systemic administration can effectively inhibit the oxidative stress response in diseases and promote the survival and regeneration of damaged parenchymal cells. The mRNA and miRNAs in MSC-EVs and MSC-Exos are the most important bioactive molecules in disease treatment, which can inhibit the apoptosis, necrosis and oxidative stress of lung, heart, kidney, liver, bone, skin and other cells, and promote their survive and regenerate.

## 1 Introduction

Mesenchymal stem cells belong to a heterogeneous cell population of stromal cells. They proliferate and differentiate *in vitro* in the form of plastic adherent cells. They can be isolated from many human tissues and differentiate into mesoderm and endoderm (1), neuroectodermal cells (2) and other embryonic lineage cells (3). Mesenchymal stem cells have been proven to have a variety of biological functions. They can interact with cells in the immune system for immune regulation, inhibit tumor necrosis factor (TNF), upregulate IL-10 (4), reduce inflammation, inhibit respiratory burst and Activate the spontaneous apoptosis of neutrophils, etc. (5). Although mesenchymal stem cells have many applications in the field of life sciences, their effectiveness is restricted by many factors. For example, the phenomenon of immune rejection of allogeneic mesenchymal stem cells (6), low persistence of curative effect of limited infusion of mesenchymal stem cells, deprivation of nutrients and growth factors and limitation of oxygen transport during mesenchymal stem cell transplantation (7), Passaged late mesenchymal stem cells trigger immediate menstrual blood-mediated inflammatory response (IBMIR) and form blood activation markers, etc. (8). In recent years, due to the vigorous development of research on exosomes derived from mesenchymal stem cells (MSC-Exos), it has been found that exosomes can avoid some of the shortcomings of mesenchymal stem cells in the treatment of various diseases.

Exosomes are spherical particles with a diameter of about 40 ~ 150 nm, which are released after the fusion of vesicles and cell membrane (9). It has lipid bilayer membrane structure, which is produced in cell culture or body fluid supernatant, such as blood, saliva, urine, breast milk, cerebrospinal fluid, bile and lymph, and can secrete exosomes (10–12). Compared with plasma membrane, exosomal membrane is harder and more stable in external environment. Exosomes carry a lot of genetic material similar to stem cells, including microRNAs (miRNAs) and mRNAs (13). In addition, exosomes contain a specific family of proteins such as heat shock protein integrins and tetrathione involved in membrane transport and fusion (14–16). It is worth noting that exosomes-based cell origin exosomes also play a unique role in cell communication (17). MSC-Exos has become the preferred treatment for many diseases and is a safe and effective stem cell-free replacement therapy (18). Exosomes are more stable and modifiable than mesenchymal stem cells and have no risk of tumor formation Due to the nanometer size and lipid bilayer structure of exosomes, exosomes can easily cross the biological barrier and enter the target organs (19). The therapeutic effect of MSC-Exos has been confirmed in a variety of diseases, including lung injury, myocardial injury, kidney injury, nerve injury, skin injury and aging (20).

**Figure 1:**
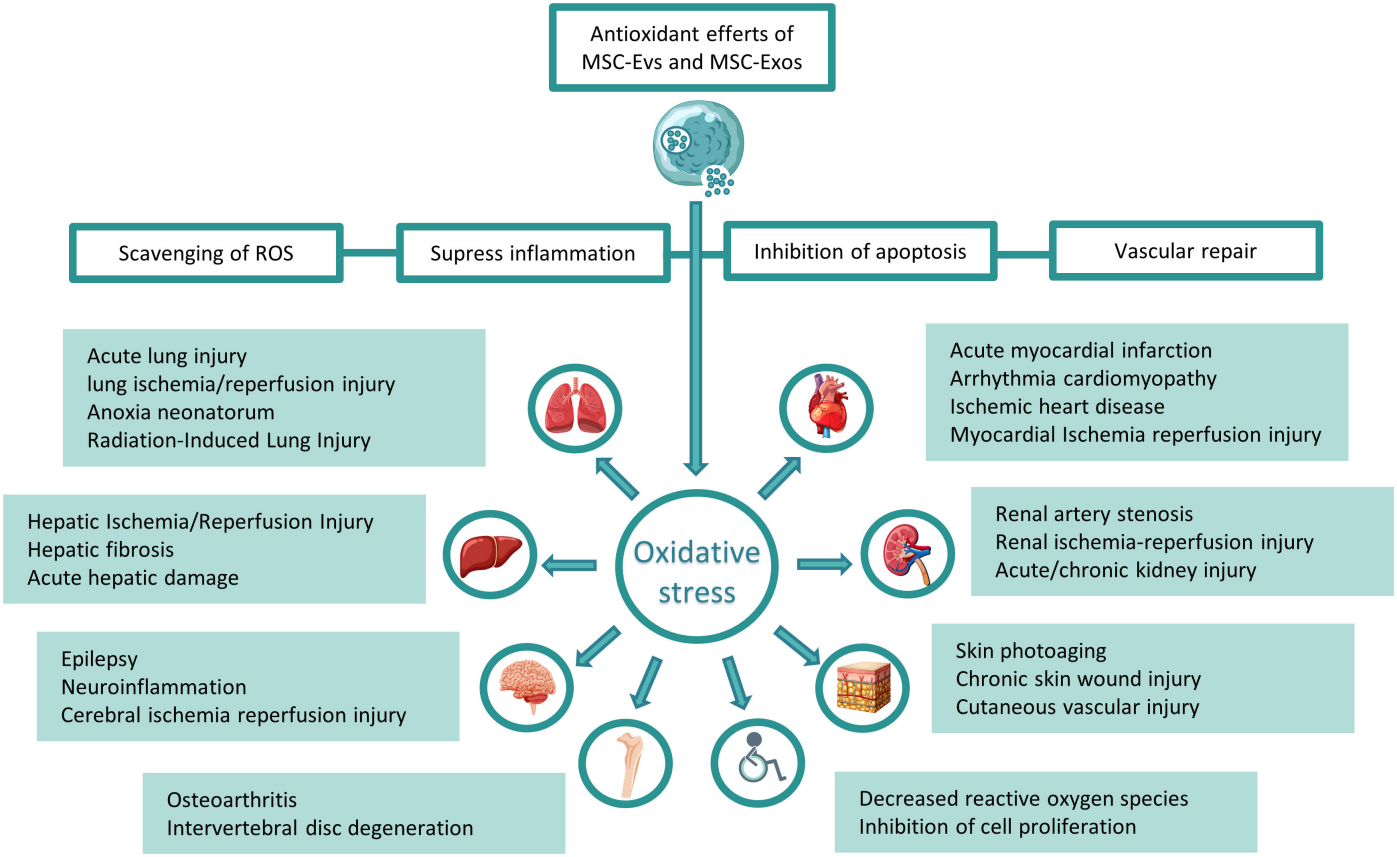
Treatment of related systemic diseases caused by oxidative stress with mesenchymal stem cell exosomes and vesicles: Mesenchymal stem cell-derived exosomes and exosomes can decrease ROS, inhibit inflammation, reduce cell apoptosis, and enhance angiogenesis, thereby treating respiratory, circulatory, and digestive systems, nervous system, motor system and other system diseases caused by oxidative stress.

When a living cell is damaged by free radicals or non-free radicals, it will obtain electrons from its molecules, which will produce a chain reaction and eventually lead to the damage of cell structure. Among these molecules, molecules from ROS (reactive oxygen species) have major biological effects, and the concept of oxidative stress is derived from this (21). Oxidative stress injury refers to the condition that oxygen and oxygen-derived free radicals exceed the natural antioxidant defense capacity of cells (22). The aggravation and prolongation of symptoms caused by oxidative stress is always a major problem in various common clinical diseases. The antioxidant activity of MSC-Exos and MSC-EVs has its unique advantages in inhibiting oxidative damage and alleviating inflammatory reaction MSC-Exos and MSC-EVs can increase calcium inflow, reduce the concentration of pro-inflammatory factors and reduce the production of ROS (23). Exosomal therapy has a looser regulatory approach than cell therapy and is considered as a “biological drug” with broad development prospects (24). In recent years, the research on exosomes is very active, and the articles on exosomes in the treatment of oxidative stress have accumulated a certain amount. This paper mainly reviews the mechanism, advantages, disadvantages and prospects of MSC-Exos and MSC-EVs derived from mesenchymal stem cells in the treatment of oxidative stress-related diseases in various systems. Based on previous studies, it details the related mechanisms, looks for the intersection of research directions, and deeply discusses the future research directions in this field (**Figure 1**).

## 2 Respiratory diseases

Therapies based on MSC-EVs or MSC-Exos have great application prospect in the treatment of oxidative stress-related lung injury. For example, acute lung injury (ALI), neonatal hypoxia-ischemia-reperfusion COVID-19 radiation lung injury, etc.

The main characteristic of ALI progression is oxidative stress response (25). The accumulation of excess pro-inflammatory factors can lead to the occurrence of oxidative stress in lung tissue, which then leads to the occurrence of ALI. At present, many drugs in the market cannot pass through the lung blood-air barrier, which leads to low curative effect, which is the main problem in treating ALI. Studies have shown that MSC-EVs can cross the blood-air barrier and other biological barriers in the lungs to enhance the therapeutic effect (26). Hyperoxia-induced lung injury can lead to the imbalance of oxidative-antioxidant system *in vivo* and lead to oxidative stress injury. The negative effects of ischemia-reperfusion are caused by the induction of inflammation and oxidative stress and the damage of cell energy metabolism, which leads to a series of harmful biological events from ion homeostasis failure to cell death (27). In severe acute respiratory syndrome (SARS) caused by SARS-associated coronavirus (SARS-CoV), the immune response to virus infection and cytokine storm play a key role in the severity of the disease (28). Cytokine storm will lead to the development of oxidative stress due to ROS produced by immune cells (29). Complex pathophysiological mechanisms indicate that severe COVID-19 is more suitable for multi-effect drug therapy than single target drug (30). Therefore, it is an innovative method to use MSC-EVs or MSC-Exos combined with COVID-19 clinical drugs.

Determining the effective therapeutic components carried by exosomes is the basis for explaining the therapeutic mechanism, including effective secretory proteins and microRNA (miRNA), etc. (31) At the same time, it is also important to further understand the therapeutic mechanism of exosomes in the lung, including antioxidant and anti-inflammatory treatment of target cells or injured tissues. miRNA is a small non-coding RNA molecule with 19-25 nucleotides that play various roles in physiology and disease progression (32). MSC-EVs have been shown to promote the repair of lung injury by delivering miR125amiR-181b and miR-126 in the field of ALI (25). Both inhalation and tail vein injection of MSC-EVs decreased the levels of pro-inflammatory cytokines IL-1 β MCP-1IL-1 α TNF α and IL-12 and increased the levels of anti-inflammatory cytokines IL-10. HE staining showed that MSC-EVs could improve pulmonary morphological changes such as alveolar wall thickening, alveolar septum congestion and inflammatory infiltration after lipopolysaccharide (LPS) stimulation (33). In addition, NF-kB is a key transcription factor, which has been proved to be a key signal factor for regulating pro-inflammatory cytokines in sepsis-induced ALI (25). The increase of pathological score showed that MSC-EVs treatment reversed the changes related to inflammatory infiltration. These studies suggest that MSC-EVs and MSC-Exos can improve the damage and pathological changes of oxidative stress-related lung diseases in the early stage and are excellent new therapeutic drugs.

### 2.1 MSC-EVs and MSC-Exos improve the related indexes of pulmonary oxidative stress

The antioxidant effects of MSC-EVs and MSC-Exos are closely related to their secreted proteins and microRNA. In exosomes-treated rat alveolar macrophages (NR8383 cells), the activity of SOD and GSH increased and the content of MDA decreased, and the effect was more obvious when the exosomes overexpressed miR-22-3p However, inhibiting FZD6 in exosomes inhibited SOD and GSH activities and increased MDA content, which indicated that miR-22-3p improved the antioxidant activity of cells through FZD6 (32). After adding MSC-EVs to raw 264.7 cells, the expression of Nrf2HO-1HMGB1 and other oxidative regulators decreased, and the number of 8-OHdG positive cells decreased. After knocking out Nrf2 in MSC-EVs and adding MSC-EVs again in raw 264.7 cells stimulated by LPS, the expression of HO-1IL-6 was up-regulated, and the expression of Nrf2Keap-1TNF-α did not change. These results indicate that knockout of Nrf2 in MSC-EVs can weaken the anti-inflammatory and antioxidant activities of EVs (33)

Transcriptome sequencing of mouse ALI model treated with MSC-EVs for 4 days showed that many genes related to immune regulation and oxidative stress were less different than those of ALI mice (34). In addition, a series of genes such as TLR4,Arg-1 and HMOX1 have been shown to be involved in immune regulation and antioxidant activity HO-1 is an antioxidant enzyme that can inhibit apoptosis, inflammation and cell proliferation to reduce cell death in ALI animal models by inducing several detoxification enzymes and antioxidant proteins (25). In summary, MSC-EVs plays an important therapeutic role in immune regulation and reduction of oxidative stress in ALI mouse model (33).

### 2.2 MSC-EVs and MSC-Exos in the treatment of lung cell injury and apoptosis induced by oxidative stress

MSC-EVs and MSC-Exos have been proved by many studies to alleviate lung pathological injury and apoptosis.

*In vitro*, the cell viability of NR8383 cells decreased and apoptosis was promoted after LPS treatment, while miR-22-3p up-regulated MSC-EVs increased cell viability and inhibited cell apoptosis FZD6 belongs to the “curl” gene family and is highly expressed in both adult and fetal lung tissues (35). Up-regulation of miR-22-3p or down-regulation of FZD6 in MSC-Exos can improve cell viability and inhibit apoptosis (32). In addition, up-regulation of miR-30b-3p of MSC-Exos in LPS-treated mouse lung epithelial cells (MLE-12 cells) also showed increased cell proliferation and inhibition of apoptosis (36).

*In vivo* experiment, the acute lung injury induced by sepsis in mice was basically normal after MSCs-EVs treatment, and the blood barrier of type I alveolar cells and endothelial cells were only slightly swollen. After exosomes treatment, the edema and bleeding of mice were alleviated (25). In the survival analysis, Kaplan-Meier survival curve showed that the survival rate of mice with cecal ligation and puncture (CLP) treated with MSCs-EVs was significantly higher (25). MiR-21-5p carried by MSC-Exos is a kind of cancer-promoting miRNA, which can effectively resist apoptosis. Ji Wei Li et al. treated mouse bone marrow-derived MSCs with hypoxia or miR-21-5p antagomir respectively to increase or decrease miR-21-5p concentration in MSC-Exos. It was found that MSC-Exos treatment weakened mouse lung ischemia-reperfusion injury in a miR-21-5p-dependent manner, effectively reduced oxidative stress-induced apoptosis and partially reduced hypoxia/reoxygenation-induced pro-inflammatory “M1” polarization of alveolar macrophages (37).

EVs can release glycosaminoglycan serum hyaluronic acid (HA) and improve energy metabolism of lung cells exposed to ischemia-reperfusion. This is particularly important in protecting against ischemic injury because HA is essential for maintaining the integrity of lung epithelial cells and inducing tissue healing and regeneration HA released by mesenchymal stem cells can trap immune cells in extracellular matrix (38–40) and prevent leukocytes from adhering to activated endothelial cells (40, 41). The impaired energy metabolism after ischemia-reperfusion injury is the main reason for the failure of ion homeostasis, which leads to cell swelling and apoptosis/autophagy activation. The treatment of MSC-EVs can restore ATP to baseline level and promote leukocyte homing in perfusion lung injury (27). These results suggest that MSC-EVs and MSC-Exos can enhance the inhibition of cell regeneration and apoptosis in oxidative stress-induced lung injury, especially miRNA in exosomes.

### 2.3 MSC-EVs and MSC-Exos in the treatment of lung inflammatory injury induced by oxidative stress

MSC-EVs and MSC-Exos play a significant biological role in anti-inflammation. ROS signal plays an important role in the occurrence and development of inflammatory injury. Excessive ROS promotes cell injury and death when the production and elimination of ROS are unbalanced (42). MSC-EVs and MSC-Exos can regulate and attenuate the inflammatory injury induced by oxidative stress in the lungs.

The expression of CD86 in mouse mononuclear macrophages was increased by LPS stimulation *in vitro*. After administration of MSC-EVs, the expression of CD86 decreased significantly, and the expression of Arg-1 increased, which indicated that macrophages polarized towards M2 anti-inflammatory direction (33).

*In vivo* MSC-EVs significantly reduced the levels of pro-inflammatory cytokines including TNF-alpha IL-1bIL-6MPO and significantly increased IL-10 levels and resulted in neutropenia in alveolar lavage fluid in a sepsis-induced acute lung injury model in mice. The phosphorylation activation of MAPK pathway is thought to play an important role in the pathogenesis of ALI and administration of MSC-EVs significantly inhibits their phosphorylation. Treatment with MSC-EVs decreased the phosphorylation level of NF-kB p65 and inhibited the increase of NF-kB p65 degradation. In addition, TLR4 also plays an important role in the regulation of inflammation and signal transduction. The decreased expression of TLR4 and its downstream signals (IL-1 β IL-6 and NF-kabb-p65) after MSC-EVs treatment also indicates that MSC-EVs can alleviate inflammation stimulated by LPS (33). These phenomena suggest that MSC-EVs play an important role in inhibiting inflammatory markers related to oxidative stress in the lungs.

### 2.4 MSC-EVs and MSC-Exos in the treatment of pulmonary vascular injury induced by oxidative stress

Vascular endothelial growth factor (VEGF) plays an important role in promoting angiogenesis and enhancing vascular permeability. Tracheal transplantation of MSC-EVs in rat lung tissue can significantly improve hyperoxia-induced angiogenesis damage and reduce the number of apoptotic cells in lung tissue (43).

Pericytes are cells that surround capillaries and veins throughout the body. Franco et al. (44) reported that pericytes can protect vascular endothelial cells from cytotoxicity. Covering vascular endothelial cells with pericyte prevents EVs from being phagocysed by vascular endothelial cells. Pericyte-dependent survival signals are forced by paracrine and autocrine circulation involving VEGF-A expression. In addition, some studies have shown that the expression of VEGF in differentiated pericyte enhances the survival of endothelial cells and the stability of microvessels (45). In general, these findings suggest that the improvement of angiogenesis of MSC-EVs is not mediated directly by phagocytosis of vascular endothelial cells, but indirectly induced by phagocytosis of pericyte by EVs. These results indicate that VEGF protein and mRNA carried by EVS are very important for angiogenesis, and the specific mechanism needs further study (43).

In conclusion, MSC-EVs or MSC-Exos play a positive role in various diseases of respiratory system. Firstly, exosomal proteins and miRNA have powerful functions, which can reach lung injury tissues through their targeting and homing properties to treat oxidative stress, inflammation and vascular injury (46). Secondly, exosomes can be used as carriers to encapsulate drugs across the air-blood barrier and alveolar epithelial-endothelial barrier to reach some places that drugs cannot reach. Finally, exosomes also show potential in disease diagnosis. Exosomes released by infected cells carry many pathogen-derived molecules and can be used as biomarkers of specific infectious factors. In addition to the frontier research directions such as exosomes and miRNAs, exosomes and autophagy, we believe that the combination of exosomes of infected cells and omics techniques to find biomarkers of various lung diseases may be the future research direction (47).

## 3 Diseases of circulatory system

It is well known that cytokines secreted by MSCs can enhance cardiomyocyte proliferation, reduce cardiomyocyte apoptosis, improve the microenvironment of damaged sites and repair damaged tissues (48).

### 3.1 MSC-EVs and MSC-Exos improve the related indexes of oxidative stress in circulatory system

Exosomes extracted from mesenchymal stem cells can improve myocardial dysfunction after hypoxia and myocardial infarction. Studies have shown that miR-182-5p carried by MSC-exos can treat myocardial ischemia-reperfusion (I/R) injury. MiR-199a-3p and miR-214 are similar to miR-182-5p in the response to I/R in rat myocardial cells, and both of them can improve the survival rate of myocardial cells and thus treat myocardial ischemia-reperfusion injury (49). At the same time, after hypoxia-induced injury, a low expression level of miR-182-5p was also observed in rat cardiomyocytes. Exosomal miR-182-5p engrafted NLRP3 inflammasome activation and cellular oxidative stress, thereby alleviating myocardial ischemia-reperfusion injury NLRP3 plays a key role in many diseases and can be activated by many types of agonists or risks. miRNA (miR-223) can bind to the 3 ‘UTR of NLRP3 to inhibit the inflammatory response caused by NLRP3 (50). On the other hand, overexpression of miR-182-5p downregulates Toll-like receptor 4 (TLR4), inactivates proinflammatory cytokines, TNF-α, and IL-6, thereby ameliorating liver I/R injury. In addition, miR-182-5p can also down-regulate TLR4 and inhibit ROS production to treat oxidative stress injury and apoptosis induced by atherosclerosis (51).

O’Brien CG et al. participated in the Seneca trial sponsored by the National Institutes of Health/National Heart Lung and Blood Institute. Peripheral blood mononuclear cells were successfully induced into induced pluripotent stem cells (IPSCs) and then differentiated into cardiomyocytes (ICMs). Using these AIC patient-specific MSCs, it was demonstrated that specific MSCs successfully reactivated iCMs after doxorubicin (DOX) injury. This effect is due to the active mitochondria carried by MSC-EVs (51).

Previous studies have shown that the re-expression of lncRNA alpha-2-macroglobulin antisense RNA 1 (Lnc RNA A2M-AS1) and Lnc RNA A2M-AS1 in myocardial infarction can significantly attenuate the hypoxia/resuscitation effect through the expression of inflammatory factor receptor IL1R2 (52). Oxygen (H/R)-induced cardiomyocyte apoptosis suggested that Lnc RNA A2M-AS1 may be involved in myocardial I/R injury.

AC16 cells were pretreated with exosomes and treated with ischemia-reperfusion. Cell proliferation was detected by CCK-8 assay, which showed that hMSCs-Exos treatment could reverse the decline in cell viability caused by ischemia reperfusion (53). Furthermore, H/R resulted in the apoptotic rate of AC16 cells, accompanied by downregulation of Bcl-2 protein levels and upregulation of Bax and Caspase-3 protein levels, which was attenuated in hMSCs-Exos treatment. hMSCs-Exos treated the content of LDH and MDA in H/R AC16 cells, but increased the content of SOD, thereby inhibiting oxidative stress. These results suggest that exosomes isolated from hMSCs can protect cardiomyocytes from H/R injury (54). It was further found that the level of Lnc RNA A2M-AS1-Exos was upregulated in AC16 cells cultured with hMSCs-Exos, alleviating H/R-induced apoptosis and oxidative stress in cardiomyocytes (54). Sánchez-Sánchez R et al. found that miR-4732-3p can induce apoptosis, ROS level and LDH activity in neonatal rat cardiomyocytes after OGD (glucose and oxygen deprivation model), prevent fibroblast migration and myofibroblast differentiation, induce *in vitro* and *in vivo* Angiogenesis. Intramyocardial injection of miR-4732-3p in exosomes in infarcted nude rats confers cardioprotection through functional and morphometric studies (55).

### 3.2 MSC-EVs and MSC-Exos in the treatment of circulatory vascular injury induced by oxidative stress

In recent years, a large number of evidences have shown that exosomes have protective effect in ischemic heart, which can alleviate myocardial I/R injury, promote cardiac regeneration and angiogenesis and inhibit fibrosis (56, 57). It has been proved that MSC-Exos overexpressing miR-486-5p can restore cardiac function after myocardial infarction in mice and non-human primates (58). Delivery of MSC-EVs containing miR-1505p in I/R rat model also alleviates poor myocardial remodeling (59). MSC-EVs-derived miR-21a5p induces cardiac protection in mice after I/R injury (60). adenovirus-transmitted miR-148a prevents ventricular remodeling in pressure overloaded mice (61). In mechanism, miR-210 was found to enhance myocardial vascularization in AMI rat model after myocardial transduction by up-regulating the expression of hepatocyte growth factor (62). Nevertheless, some miRNA in MSC-EVs may still be harmful to heart function, and the strategy of eliminating specific packaged miRNA molecules has been proved to improve the anti-apoptosis and angiogenesis of EVs (63).

Overexpression of Notch1 intracellular domain (N1ICD) in MSC-EVs can prevent apoptosis of CMS (cardiomyocytes) and promote cardiac angiogenesis under oxidative stress and ischemia injury. Notch plays an important role in cardiac repair after myocardial injury, Overexpressed MSC-EVs of N1ICD have good curative effect on angiogenesis of ischemic myocardium, proliferation of CMS (myocardial cells), improvement of cardiac function and fibrosis, Notch1 is strong for heart (64).

### 3.3 MSC-EVs and MSC-Exos in the treatment of cardiomyocyte apoptosis induced by oxidative stress

Mesenchymal stem cells can secrete a large number of soluble factors to promote myocardial cell proliferation, reduce apoptosis, improve ischemic microenvironment and mobilize endogenous cardiac stem cells by paracrine. However, studies have shown that although exogenous mesenchymal stem cells prefer to homing to myocardial ischemia sites, their survival rate in infarct areas is low (65). MSCs-Exos mediates cell-to-cell communication through horizontal transfer of bioactive RNA molecules and proteins (66). It is important that exosomes have good stability without the risk of chromosome loss and poor immune responses are rare (48). Therefore, MSCs-Exos is considered as an ideal drug delivery carrier and has great prospects in the treatment of oxidative stress-induced cardiomyocyte apoptosis.

### 3.4 MSC-EVs, MSC-Exos and autophagy of myocardial cells

Autophagy is involved in regulating the metabolic balance between synthesis, degradation and reuse of cellular substances. Both autophagy defect and overactivation will cause damage to homeostasis to some extent (67). Bafecycin A1 (Baf-A1) inhibited autophagy, and observed the effect of exosomes on autophagy. Compared with pure H_2_O_2_-induced autophagy, the expression of Beclin-1 and LC3B-II in exosomes + H_2_O_2_-treated cells increased and the expression of P62 decreased, while the protein levels of LC3B-IIBeclin-1 and P62 in H_2_O_2_ + exosomes + Baf-A1-treated cells were higher than those in H_2_O_2_ + exosomes and H_2_O_2_-treated cells. Therefore, autophagy induced by MSCs-Exos may be an important mechanism of cell protection after H_2_O_2_ (48). Autophagy is known to be associated with many pathways involving MAPK/mTOR and Akt/mTOR. Enhanced autophagy after hypoxia or ischemia injury has myocardial protection. Excessive ROS production in post-ischemia reperfusion stage can lead to autophagy. Matsui et al. reported that activation of MAPK pathway can induce autophagy, but activation of Beclin-1 pathway can induce autophagy, which may lead to cell death during reperfusion after ischemia (30). Therefore, ROS-induced autophagy is multifaceted (68).

At present, the research on the application of MSC-EVs and MSC-Exos in circulatory system diseases mainly focuses on microRNA. Numerous studies have found which microRNA in exosomes has a therapeutic effect on circulatory system diseases through sequencing technology. For example, miR-182-5p of MSC-Exos can improve myocardial I/R injury through the expression of GSDMD (51), miR-21a5p derived from MSC-EVs induced cardioprotection in mice after I/R injury (60), etc. Exosomes are natural carriers of bioactive molecules, and using them as carriers of drugs or siRNA to control gene expression and accelerate disease recovery may be a research hotspot in the future of exosomes in the circulatory system (69).

## 4 Diseases of digestive system

Oxidative stress is the main pathogenic phenomenon peculiar to liver diseases, which may lead to common liver diseases (70). Under normal circumstances, hepatocytes can balance the advantages and disadvantages of oxidative stress, control the level of oxidative stress within a reasonable range, and have the ability to regulate the balance of oxidants and antioxidants. However, due to the damage caused by toxins, there will be an imbalance between these particles. Oxidative stress is caused by mitochondrial dysfunction of hepatocytes, which leads to ROS production. This will not only induce irreversible changes in lipid protein and DNA content, but also regulate the pathway of controlling normal biological function (71). Studies have shown that MSCs-Exo can produce beneficial effects in animal models of various liver diseases, including liver injury, liver fibrosis, ischemia-reperfusion and so on.

### 4.1 MSC-EVs and MSC-Exos improve the related indexes of oxidative stress in liver injury

MSC-Exos can be used as an antioxidant to oxidative stress in mice with liver injury. *In vitro*, Hiroaki Haga et al. evaluated the effects of MSC-EVs on ROS production and NF-kB activity in normal mouse stem cells induced by H_2_O_2_ ROS activity was observed after 1 hour of H_2_O_2_. This activity was significant after 24 hours pre-incubation with MSC-EVs. The same NF-kB activity was also detected by MSC-EVs. Therefore, the results show that MSC-EVs can regulate the response to oxidative stress in liver IRI (72). GPX1 is an antioxidant that can induce oxidative stress injury induced by H_2_O_2_ to promote cell survival. When GPX1 in hucMSC-Exos is knocked out, the antioxidant activity of exosomes indicates that GPX1 is an important factor in hucMSC-Exos-mediated antioxidant activity and liver protection. Inducing increased GPX1 activity can induce liver injury and activate mitochondrial apoptosis pathway in hepatocytes (70). *In vivo*, hucMSC-Exos significantly inhibited the activation of oxidative stress products 8-OHdG and SOX9 in CCl4-induced liver tumor model. In CCl4-induced acute liver injury model, 8-OHdG also had more obvious antioxidant and liver protection effects than biphenyl ester (DDB) treatment, and hucMSC-Exos was a more effective antioxidant than DDB (73).

### 4.2 MSC-EVs and MSC-Exos in the treatment of liver inflammatory injury induced by oxidative stress

Macrophages and Kupffer cells are involved in regulating liver inflammation and hepatocyte death in liver. Studies have shown that exosomes can promote disease recovery by expressing inflammatory factors (72). MSC-EVs can reduce ROS production and overexpression of inflammatory cytokines such as IL-6 and IL-1B due to activation of the NF-kB signaling pathway. In addition, MSC-EVs can inhibit inflammation in liver through NLRP12, which is a negative regulator of inflammatory activity *in vitro* immune system and others, and plays a role through attenuated NF-kB (72).

### 4.3 MSC-EVs and MSC-Exos in the treatment of hepatocyte apoptosis induced by oxidative stress

The results of clinical studies show that HUCMSCs transplantation can improve the blood supply of hepatocyte extensive necrosis and other clinical symptoms in decompensated cirrhosis (70). The levels of ALT and AST, the markers of hepatocyte injury, and the levels of Caspase-3 and Bcl-2, the activity of Bax and the anti-apoptosis protein increased after treatment with hucMSC-Exos. These results suggest that hucMSC-Exos treatment can improve liver I/R injury (74). In acetaminophen (APAP) and hydrogen peroxide (HP) induced hepatocyte injury models treated with hucMSC-Exos, cell activity increased, necrosis and apoptosis decreased, LDH activity decreased and ROS decreased. In CCl4-induced acute liver injury cells treated with hucMSC-Exos, Bax and activated caspase 3 expressed TUNEL positive cells can inhibit hepatocyte degeneration and hepatic lobules at the same time, and even partially save the life of mice in acute liver injury model. It is confirmed that hucMSC-Exos can induce acute extensive liver injury induced by CCl4 (73). ERK1/2 phosphorylation and Bcl-2 expression induced by glutathione peroxidase 1 (GPX1) hucMSC-Exos in mice. The phosphorylation of I-Kappa-B Kinase b (IKKb) and NF-kB was inhibited 24 hours after hucMSC-Exos treatment. The expression of Casp-9 and Casp-3 was inhibited HucMSC-Exos inhibited the I-Kappa-B Kinase b (IKKb) NF-kBcaspase 3 pathway and pNF-kB nuclear translocation in CCl4-injured hepatocytes in a dose-dependent manner and induced Bcl-2 expression and ERK1/2 phosphorylation to reverse oxidative stress-induced apoptosis (70).

### 4.4 MSC-EVs and MSC-Exos regulate iron death in the treatment of liver injury

Iron death is a regulatory cell death caused by lipid peroxidation. Iron death is very important for preventing various liver diseases, including liver fibrosis. HSCs iron prolapse has become the target of inhibiting liver fibrosis (75). Benzyl chloride 1 (BECN1) is the key regulator of iron sag (76). It was found that BECN1 enriched down-regulated GPX4 in both MSCs-EVs and MSC-Exos, which contributed to iron sagging, which was necessary to activate HSCs (77). An increase in BECN1 was detected after MSC-Exos treatment, and a decrease in GPX4 and alpha-SMA (HSCs-activated markers) was also found in fibrotic mouse livers and collagen deposition. Therefore, MSC-Exos containing BECN1 can induce BECN1/GPX4-mediated iron prolapse and activation of HSCs in mouse fibrotic liver. When BECN1 was knocked out, ROS expressed by GPX4 produced mitochondrial membrane potential. Therefore, BECN1 can induce iron poisoning by down-regulating GPX4. BECN1 overexpression can produce ROS and decrease mitochondrial membrane potential *in vivo* experiments also showed the same results (77).

In the current report, MSCs-EVs and MSC-Exos were not only able to down-regulate inflammatory factors and oxidative stress indicators in diseases of digestive system, but exosomes were also found to regulate iron death in liver fibrosis. Iron death, a novel type of programmed cell death, occurs in a wide range of injured cells, and there are numerous research points that can be explored, which deserve to be deeply investigated.

## 5 Urinary system disease

Combination of MSC-EVs and MSC-Exos transport with renal artery revascularization can improve renal function and structure and narrow oxidative stress, apoptosis, fibrosis and microvascular remodeling (78, 79). Renal artery stenosis (RAS) is very common in patients with chronic kidney disease. RAS patients are prone to renovascular hypertension and progress to end-stage renal disease (80). MSC-EVs have been shown to alleviate renal inflammation and microvascular damage and improve hemodynamics and function beyond stenosis in porcine RAS (81, 82). This suggests that MSC-EVs are effective in preserving stenosis.

### 5.1 MSC-EVs and MSC-Exos regulate renal oxidative stress-related diseases through mitochondria

Mitochondria regulate many functions of renal cells, including redox state, survival, proliferation and death. The damage of mitochondrial structure and function is often accompanied by oxidative stress, which is mainly due to the production of superoxide (83) and H_2_0_2_ by complex I and III, which damages several components of mitochondria and forms a vicious circle of mitochondrial damage and oxidative stress. Mitosis can improve the revascularization results of experimental (84) and clinical RAS and protect renal function. Studies have shown that MSC-EVs and MSC-Exos play an important filamentous protective role in stenotic kidney, which improves mitochondrial density and mitochondrial swelling (85).

The important role of mitochondria in mammalian cells is to produce ATP through OXPHOS (86). Loss of a nuclear-encoded mitochondrial protein, TFAM, causes mtDNA depletion and OXPHOS (87, 88). After treatment of HK-2 with H_2_0_2_, the basal respiration rate, the maximum respiration rate, the ATP production respiration rate and the standby respiration capacity level all made MSC-EVs reverse these phenomena. Therefore, MSC-EVs can function as mitochondria (89).

Faisal A Alzahrani and others found that MSC-Exos significantly decreased the levels of MDA,HIF-1 α, mRNA and NADPH oxidase 2 (NOX2) protein and increased the levels of three antioxidant enzymes and HO-1 mRNA. The changes of oxidative stress or antioxidant related parameters were more prominent after ischemia. This indicates that MSC-Exos can play a role by inducing antioxidant activity and inhibiting oxidative stress in injured kidney tissue (90). Pallavi Bhargava et al. (86) used renal I/R injury model to evaluate the therapeutic effect of MSC-EVs. The results showed that the rate of renal tubular necrosis, the expression of KIM-1 and the number of apoptotic cells in I/R mice, And I/R mice showed higher levels of renal cytokines mRNAs (IL-6,IL-1 β and ICAM1) and serum cytokines (TNF-α and TWEAK) (91, 92). MSC-EVs treatment can reverse the levels of these cytokines.

### 5.2 MSC-EVs and MSC-Exos regulate related indexes of kidney oxidative stress

It is well known that most of the beneficial effects of MSCs are mediated by their exosomes containing miRNA, mRNA and LncRNA. These exosomal RNA are responsible for intercellular communication through which RNA-based information is transmitted to recipient cells (93). Interestingly, when exosomes were pretreated with RNase, the ameliorative effect of exosomes on renal ischemia-reperfusion injury was eliminated, which means that exosomal RNA has an ameliorative effect on oxidative stress in renal injury (94). In addition, Rafael S Lindoso et al. (95) reported that the ameliorative effect of MSCs-Exos on Renal ischemia-reperfusion injury and metabolic syndrome is related to the expression of some miRNA involved in apoptosis and hypoxia, which means that exosomes can improve their effects by post-transcriptional targeting of some genes in cells by exosomal miRNA Melatonin (Mel) preconditioning may induce MSCs to produce exosomes with higher expression of RNA vectors, which induce renal repair by inhibiting certain molecules involved in oxidative stress cell apoptosis and inflammation (90).

### 5.3 MSC-EVs and MSC-Exos in the treatment of apoptosis induced by oxidative stress

Renal ischemia-reperfusion injury may accelerate the development of chronic kidney (CKD). Renal ischemia-reperfusion injury releases free radicals and mitochondrial function induces apoptosis and inflammation (96, 97). The degree of renal function was evaluated by detecting BUN in plasma and creatinine in serum BUN and creatinine levels decreased sharply after 4 weeks of renal ischemia-reperfusion injury, but they were still higher than normal renal tissue. These elevated levels improved significantly after MSC-Exos preconditioning and creatinine levels returned to normal at 4 weeks. Therefore, the renal dysfunction after renal ischemia-reperfusion injury is relieved after MSC-Exos treatment, which means that MSC-Exos has broad prospects in the treatment of CKD (90). Exosomes can improve renal ischemia-reperfusion injury by interfering with apoptosis of renal cells. This inhibition of apoptosis can be evaluated by measuring the activity of caspase-3, the final marker of apoptosis. qPCR results showed that apoptosis markers Bax,PARP1 and caspase-3 were up-regulated and anti-apoptosis marker. Bcl2 was down-regulated in renal ischemia-reperfusion injury rats Bax,PARP1 and caspase-3 mRNA levels, and Bcl2 mRNA levels were significantly increased after treatment with MSC-Exos. These results suggest that the ameliorative effect of MSC-Exos on renal ischemia-reperfusion injury is related to the inhibition of apoptosis in damaged kidneys (90). In addition, MSC-Exos had similar anti-apoptosis effects on ischemia-reperfusion injury (98, 99) cisplatin-induced acute kidney injury (100, 101) and glycerol-induced acute kidney injury (102). This anti-apoptosis effect is also related to enhanced proliferation of renal tubular epithelial cells and improved renal function and structure (90).

### 5.4 MSC-EVs and MSC-Exos in the treatment of vascular injury induced by oxidative stress

Transcription factor Sox9 plays a key role in renal development, and its dysfunction can lead to severe renal dysplasia (103). It is reported that the improvement of AKI by MSC-Exos is mediated by up-regulating the expression of Sox9 in renal tubular cells (104). Injection of MSC-Exos in renal ischemia-reperfusion injury rats induced the expression of various angiogenic factors, resulting in the improvement of renal function (94, 105), suggesting that these angiogenic factors may be involved in exosomal-induced renal repair. Studies reveal the important role of bFGF, HGF and Sox9 in renal tubular regeneration after renal ischemia-reperfusion injury (106). The expression of these regeneration markers was also induced after treatment with MSC-Exos (104).

MSC-EVs and MSC-Exos have a dual role including regeneration and reduction of inflammation and oxidative stress in urinary tract diseases. It is rich in growth factors that can provide nutrients. At the same time, *in vitro*, mesenchymal stem cells can transfer many cytokines with anti-apoptosis, anti-inflammatory angiogenesis and immunomodulation properties into conditioned medium to promote the recovery of model animal diseases effectively. However, the safety and ethics of this therapy should be further explored and corrected in order to try to apply it to clinical practice (107).

## 6 Diseases of nervous system

Stem cell therapy shows great prospect in nervous system diseases. However, stem cell therapy is limited by its safety ethics or national legislation. Many evidences show that MSC-Exos and MSC-EVs are more effective than their parent cells in the treatment and recovery of nervous system diseases (108). EVs have many biological characteristics, including crossing the blood-brain barrier and the ability to resist freezing and thawing, which is beneficial for EVs to play a therapeutic role in nervous system defects (109). EV carries a variety of complex RNA and protein. EV has a good ability to regulate oxidative stress pathophysiology and immune response in nervous system diseases. Especially, the ability of miRNA transfer to target cells mediated by EVs plays a key role in antioxidant activity (110) and MSCs pretreated with H_2_O_2_ have better antioxidant activity (111). Studies have shown that EVs contain a series of up-regulated antioxidants miRNA, such as miR-215-5p,miR-424-5p,miR-31-3p,miR-193b-3p and miR-200b-3p. This indicates that exosomal miRNA plays an important role in antioxidant stress (112).

### 6.1 MSC-EVs and MSC-Exos improve the indicators related to oxidative stress in the nervous system

Seizures have a disproportionate impact on patients with traumatic brain injury and stroke (113). ROS overproduction caused by oxidative stress is an important pathophysiological mechanism in human epilepsy. Oxidative stress in epilepsy-causing hippocampal neurons can lead to neuronal apoptosis, cell loss and mitochondrial function. Therefore, targeting the changes in the pathophysiology of oxidative stress in the hippocampus has a significant improvement effect on the disease (114). Electrophysiological disturbances are often manifested in neuronal function (115), depolarization responses lead to spontaneous firing and AP numbers, and input resistance can lead to impaired excitability of neuronal cells (116), especially CA1 pyramidal neurons, in Oxidative stress is particularly vulnerable during epilepsy. Improvement of neuronal membrane excitability after EVs treatment, suggesting the ability of MSC-EVs to restore hippocampal electrophysiology in cellular and animal models.

SAMPs are involved in oxidative responses and cellular homeostasis, including iNOS, HMGB1, HO-1, and Nrf2 (117). MSC-EVs treatment resulted in improvement of SAMPs, suggesting that MSC-EVs have excellent therapeutic effect on H_2_O_2_-induced oxidative neuronal injury. Furthermore, There are two types of glutamate receptors that control oxidative stress levels through mediated signal transduction. Glut 1 is also strongly associated with the proliferation and death of neurons after the cell is stimulated (117). Reversal of the expression of SAMPs, AMPA, and Glut1 after EVs treatment indicated that MSC-EVs attenuated seizure-induced oxidative stress in the mouse hippocampus.

Nrf2 is closely related to antioxidant, and the antioxidant effect of Nrf2 enables it to play a neuroprotective role in epilepsy and other neurological diseases through targeted therapy. The function of NRF2 is closely related to KEAP1, which plays a coordinated role in the expression of several target genes, such as NADPH quinone oxidoreductase 1(NQO1) and heme oxygenase 1(HO-1), which encode antioxidant mediators and have protective effects against hippocampal neuronal damage caused by seizures (118). MSC-EVs are rich in antioxidant miRNAs, and knockdown of Nrf2 abolished the antioxidant capacity of MSC-EVs against epilepsy-induced hippocampal injury, suggesting that the Nrf2 defense system is involved in the antioxidant effect of MSC-EVs in epilepsy (112).

Qiang Luo et al. pretreated hippocampal neurons with 10 μg MSC-EVs, and then with 100 μM H_2_O_2_, MSC-EVs pretreated the activities of FRAP, CAT, SOD and GSH-PX. Furthermore, ROS production in hippocampal neurons of H_2_O_2_ was assessed by flow cytometry, and a significant rate of ROS production was detected in the group pretreated with MSC-EVs. Experiments showed that H_2_O_2_ led to increased expression of 8-OHdG (DNA damage marker), 4-HNE (lipid peroxidation marker) and DT (protein oxidation marker), and EVs treatment significantly enhanced the expression of these markers, and immunofluorescence staining confirmed that MSC-EVs can process and repair DNA damage. These results suggest that MSC-EVs have a strong antioxidant capacity in hippocampal neurons in response to H_2_O_2_ (112).

Through miRNA sequencing technology, find and identify substances that may have antioxidant potential in MSC-EVs. Comparing the differentially expressed miRNAs between MSC-EVs and H_2_O_2_-derived MSCs-EVs, many miRNAs were found to be upregulated in H_2_O_2_-derived MSCs-EVs, such as miR-215-5p, miR-424-5p, miR-31-3p, miR- 193b-3p and miR-200b-3p (119). GO classification showed that exosomal miRNA target genes were closely related to antioxidant active molecules. miRNA transfection revealed that miRNA inhibitors reduced oxidative stress-induced 8-OHdG concentrations in hippocampal neurons. This suggests that these miRNAs in MSC-EVs exert an antioxidant effect on H_2_O_2_ in hippocampal neurons (112).

Calcium, as an intracellular messenger, is ubiquitous in oxidative stress (120). mitochondria are important organelles in the control of calcium homeostasis (121). MSC-EVs can restore seizure-induced hippocampal neuronal morphological changes and mitochondrial function. The expression of TOM20, FIS1, and COXIV after EVs treatment indicated that MSC-EVs could restore mitochondrial/fusion and respiratory chains, so calcium and mitochondrial changes are critical for maintaining the stability of neuronal function (122). These results indicated that MSC-EVs improved calcium transients and mitochondrial function in primary cultures in H_2_O_2_.

The MWM test, which measures cognitive ability, found that MSC-EVs-treated seizure mice had shorter escape latencies. These data suggest that MSC-EVs treatment promotes functional reconstitution of hippocampal neurons during epileptic chronic seizures (123). Qiang Luo et al. pretreated hippocampal neurons with MSC-EVs to induce the uptake of nanoparticles, and then used H_2_O_2_ cells to induce oxidative stress. The results showed that the activities of various antioxidant enzymes decreased and excessive ROS production (124). It is evident that MSC-EVs have a significant antioxidant effect on seizure-induced neuronal damage by reversing H_2_O_2_-induced oxidative stress (112).

### 6.2 MSC-EVs and MSC-Exos treat inflammatory injury of nervous system induced by oxidative stress

Astrocytes play an important role in the formation of blood-brain barrier. They can produce and express neurotransmitters and some neurotransmitter receptors In addition, astrocytes can biotransform exogenous compounds and help regulate ionization around neurons. Studies have shown that astrocytes can be activated by inflammation or ROS (125). Sexual astrocyte activation can enable mitochondrial function (126). There is an extensive interaction between Nrf2 and NF-κB, which has been shown to be involved in the regulation of transcriptional, anti-oxidative, and anti-inflammatory pathways, regulation of NRF2 and NF-κB signaling pathway can reduce some inflammatory factors and improve the function of astrocytes (127).

MSC-Exos has a good therapeutic effect on inflammatory astrocytes and can improve a series of diseases caused by inflammatory astrocytes (128). MSC-Exos can enhance the cytotoxicity of astrocytes induced by LPS; The markers of reactive astrocyte proliferation, such as GFAP,C3,CD81 and Ki67, increased significantly after MSC-Exos treatment of inflammatory astrocytes. TNF α and IL-1 β in culture medium decreased after MSC-Exos treatment of LPS-induced astrocytes. These results indicate that MSC-Exos has a good effect on inhibiting LPS-induced decrease of cytotoxic astrocyte proliferation and inflammatory response (127).

Panpan Xian et al. used calcium imaging to study calcium changes in primary cultures of astrocytes. The data revealed that different groups of astrocytes had different fluorescent properties. MSC-Exos treatment of LPS-induced hippocampal astrocytes significantly enhanced their Ca^2+^ influx, and LPS resulted in faster changes in response rise time and decay time. It is worth noting that MSC-Exos has a significant therapeutic effect on astrocyte Ca^2+^ oscillation rate and mitochondrial dysfunction in patients with LPS (129).

In conclusion, mesenchymal stem cell exosomes can reduce inflammation and oxidative stress in nervous system diseases. Exosomes are closely related to the pathogenesis of central nervous system diseases, so exosomes have the potential to become unique biomarkers of nervous system diseases. In addition, exosomes can cross the blood-brain barrier, making them a new candidate for drug carriers for nervous system diseases.

## 7 Skin tissue trauma and repair

With the development of regenerative medicine, autologous mesenchymal stem cells can be cultured *in vitro* and then injected into vivo to promote the regeneration and repair of damaged tissues (130). More and more evidences show that exosomes have excellent therapeutic effects in various disease models besides stem cells. Exogenous skin aging is usually caused by various chronic injuries such as drinking, smoking and ultraviolet radiation. Cause skin aging based on the important role of ROS in photoaging. Protect skin from photoaging by producing ROS or inducing antioxidant defense (131, 132). MSC-Exos can inhibit oxidative damage of H_2_O_2_ keratinocytes, improve antioxidant activity, reduce oxidative reactivity and improve abnormal calcium and mitochondrial changes induced by oxidative stress. Subcutaneous injection of MSC-Exos can alleviate ultraviolet-induced skin tissue damage and inflammatory reaction in mice, inhibit cell proliferation and collagen deposition in skin of mice irradiated by ultraviolet radiation, alleviate oxidative damage of mice irradiated by ultraviolet radiation, improve antioxidant activity and alleviate oxidative reaction in mice. All in all, exosomes can promote wound healing and recovery through oxidative stress in various ways in the treatment of skin injury and photoaging (23, 132).

### 7.1 MSC-EVs and MSC-Exos in the treatment of photoaging injury induced by oxidative stress

It is well known that DNA damage is a marker of oxidative stress. Skin cells will produce ROS and DNA damage after oxidation (133). The results showed that MSC-Exos treatment could prevent ROS formation and DNA damage induced by oxidative stress in mouse keratinocytes.

R. S. Stern demonstrated that both MSC-EVs and Fb-EVs could proliferate cells and prevent cell cycle arrest induced by UVB and intracellular ROS levels induced by UVB radiation. In addition, the expression of MMP-1, the expression of Col-1, the expression of MSC-EVs and the enhancement of antioxidant activity of Fb-EVs in senescent cells after EVs treatment may be related to the up-regulation of GPX-1 gene expression (134, 135). Oxidative stress can lead to inflammation and inflammation can also induce subsequent oxidative damage (136). S. Candel et al. observed that MSC-Exos injection induced the expression of pro-inflammatory cytokines (TNF α IL-1 β and IL-6) in mouse skin after ultraviolet irradiation, which means that MSC-Exos alleviated inflammatory reaction and oxidative damage in mice exposed to ultraviolet irradiation (137).

MSC-EVs can transfer MSC-EVs through GPX-1 protein to achieve antioxidant effect. Higher levels of GPX-1 can be observed in EVs treated cells (70, 138). To elucidate the mechanism of EVs-dependent ROS, the expression of antioxidant protein GPX-1 after EVs treatment, but the expression of SOD1, SOD2 and catalase was not (139).

Keratinocytes are the main constituent cells of the epidermis and form an important skin barrier to prevent damage caused by ultraviolet radiation, water loss, pathogens, fungi and viruses (140). Studies have shown that Nrf2 is very important in regulating cell homeostasis, including antioxidant proteins, detoxification enzymes, drug transporters and many cytoprotective proteins (141). Regulation of Nrf2 pathway of keratinocytes to external oxidation is a promising treatment strategy for skin photoaging injury. The results of M. Schafer et al. showed that the recovery effect of MSC-Exos after oxidation was related to the down-regulation of Nrf2. By knocking down Nrf2, we can explore the detailed mechanism of MSC-Exos activity on oxidative keratinocyte reactivity (141). The results of Wang T et al. Show that MSC-EXOS can improve oxidative stress in both *in vivo* and *in vitro* experiments. Exosomes can improve oxidative stress injury of H_2_O_2_ pretreated keratinocytes at cellular level. Exosomes at animal level can improve DNA damage and mitochondrial changes of mouse skin after ultraviolet radiation. Exosomal therapy enhances the antioxidant capacity of skin as shown by iron ion antioxidant capacity and enhances the activity of glutathione POD or superoxide dismutase in cell and skin damage induced by oxidative stress. Therefore, MSC-Exos may be used as a potential skin nano-therapeutic agent to treat skin diseases or disorders caused by oxidative stress (23).

### 7.2 MSC-EVs and MSC-Exos in the treatment of chronic wound injury induced by oxidative stress

MSC-EVs not only has a broad prospect in the study of skin photoaging, but also has a good therapeutic effect on chronic skin injury. Diabetic ulcer is a chronic trauma characterized by an inflammatory state of hypoxia and undernutrition caused by elevated blood glucose levels followed by an elevated hypoxia of oxidative stress leading to the death of fibroblasts and other skin cell types (142, 143). Parvaiz A Shiekh et al. pretreated HDFs with EVs for 6 hours and then put them in hyperglycemia in order to study the cell survival of HDFs (fibroblasts) under hyperglycemia (143). All EVs were able to protect HDFs from cytotoxic hyperglycemia at least 24 hours after treatment until at least 72 hours after determination. Interestingly, the results are even better than those of the positive control group-complete medium. They found that both AT-MSCs and HF-MSCs can oxidative stress HDFs metabolism and activity under hyperglycemia (144).

### 7.3 MSC-EVs and MSC-Exos in the treatment of skin vascular injury induced by oxidative stress

The biology of angiogenesis includes the proliferation and migration of endothelial cells and angiogenesis. New blood vessels can supply oxygen and nutrition to the wound site, so the formation of new blood vessels can determine the effect of chronic wound healing (145, 146). Many studies have shown that inflammatory reaction of oxidative stress tissue leads to stagnation of wound healing. In addition, limited vascular function and angiogenesis will cause hypoxia in chronic injuries and lead to prolonged wound healing (147). Xiao X et al. made wound models and performed healing operations on 8-week-old and 64-week-old mice. The results showed that the healing ability of old mice was weaker than that of young mice. Quantitative measurements showed that MSC-EVs had a high level of reepithelization and a low level of scar formation. The quantitative analysis of neovascularization density confirmed the beneficial effect of MSC-EVs on wound vascular reconstruction (148).

MSC-EVs play an important role in regulating functional recovery and treating wound healing. MiR-146a and Src play an important role in promoting angiogenesis and wound healing, thus dephosphorylating Src (148). Next-generation mRNA sequencing and proteomics showed that EVs contain many angiogenic genes and proteins including growth factors, nuclear receptors, adhesion molecules, protease inhibitors, matrix protein transcription factors and other factors involved in angiogenesis, suggesting that MSC-EVs have important angiogenic potential (149).

### 7.4 MSC-EVs and MSC-Exos for skin aging induced by oxidative stress

The pursuit of eternal youth to resist aging is the lifelong pursuit of human beings. Using MSC-EVs to reduce oxidative stress to achieve anti-aging effects, this research direction has become the focus of many scholars, and many research results have been achieved. H_2_O_2_ reduced the expression of skin moisture-related mRNAs (aquaporin-1 and aquaporin-3) and hyaluronic acid, while MSC-Exos reversed these effects, and H_2_O_2_-induced cellular senescence was also reproduced in fibroblasts. Matsuoka T et al. found that over time, the downregulation of SIRT1 leads to the acetylation expression of p53, thereby inducing the expression of p21, a downstream molecule of p53, delaying the cell cycle and leading to cell senescence. MSC-Exos enhanced these transduction systems, effectively blocking the increase in intracellular β-galactosidase activity and the accumulation of ROS (150).

The anti-aging effect of MSC-EVs and its mechanism are still unclear, especially the effect on endothelial cell (EC) senescence. Xiao X et al. investigated the *in vitro* effects of MSC-EVs on oxidative stress-induced aging of human umbilical vein endothelial cells (HUVEC), as well as the *in vivo* effects on natural aging and diabetic mouse wound healing models (151). In addition, they investigated its molecular mechanism using miRNA sequencing and phosphokinase antibody arrays. It is suggested that MSC-EVs can be used as nanotherapeutics through the miR-146/Src pathway. In senescent HUVECs, MSC-EVs treatment prevented senescence-induced functions and promoted angiogenesis, cell migration and proliferation ability, mitochondrial function, and ROS levels (148).

Src kinase family is inextricably linked with aging. Senescence can activate the Src kinase family, which leads to oxidative stress, lipid peroxidation and DNA strand breakage, and finally leads to fatal damage to cells. Studies have shown that Src family inhibitors (PP2) can completely block H2O_2_-induced EC_S_ aging. MSC-Exos has the same inhibitory effect as PP2. MSC-Exos can prevent senescence by inhibiting the activation of Src (152).

Studies by Zou et al. have shown that H_2_O_2_ can induce senescence in HUVEC and human aortic myocytes by up-regulating the alternative splicing body, Oct4A (153). H_2_O_2_-induced EC senescence induces a DNA damage response that activates p53 and p16, two important cell cycle regulatory pathways (154). High glucose can induce the senescence of HUVECs through the expression level of mitochondrial sirtuin (SIRT3), the expression of SA-gal, and the tube-forming ability of HUVECs (155). Oxidative stress is involved in the pathogenesis of diabetic vascular abnormalities, inducing premature senescence through DNA damage, and streptozotocin (STZ)-induced diabetes can induce senescence in ECs (156).

The miRNAs carried by MSC-EXOS has a significant effect on the treatment of cell senescence and the promotion of angiogenesis. Studies have shown that four miRNAs, miR-146a-5pmiR-34b-3pmiR-28-3p and miR-412-5p, play a promoting role in the treatment of aging ECs (157). High expression of miR-146a in MSC-EVS, when miR-146a inhibitor was used, the effect of MSC-EVS on aging disappeared. In addition, Xiao X et al. found that miR-146a can inhibit Src phosphorylation and its downstream target cavelin-1, thus inhibiting aging (148).

A large body of evidence shows that ROS can induce or accelerate ECs senescence at multiple subcellular levels. In cultured senescent HUVECs, MSC-EVs treatment prevented oxidative stress-induced ROS formation and DNA damage. Mitochondria are not the main source of ATP in ECs, but as ROS organelles, play an important role in the response of cells to pairs (158) and maintain the homeostasis of ECs (158). Experiments showed that aging-induced mitochondrial function of HUVECs could be improved by MSC-EVs treatment. Taken together, MSC-EVs have a comprehensive rescue effect on aging-induced endothelial cell function.

MSC-EVs and MSC-Exos have excellent therapeutic effects in both acute and chronic skin injuries. Exosomes can improve various functions caused by skin aging by inflammation and oxidative stress in skin injury. In recent reports, some scholars began to explore the effects of MSC-EVs and MSC-Exos on iron death and copper death induced by skin injury, which may be a research hotspot in the treatment of skin injury with exosomes in the future.

## 8 Diseases of motor system

ROS is involved in regulating many chondrocyte activities such as cell proliferation and matrix remodeling (159). Osteoarthritis (OA) is a joint disease characterized by cartilage degeneration and low-grade synovitis In damaged joints, chondrocyte homeostasis is gradually increased with the gradual increase of oxidative stress (160). MSC-EVs treatment of OA joint cells can down-regulate inflammatory factors and increase the synthesis of extracellular matrix of chondrocytes (160). Low levels of ROS play an indelible role in various physiological processes, but the formation of ROS can lead to tissue damage. The endogenous mechanism of MSC-EVs is activated by oxidative stress and can offset the influence of ROS. Therefore, oxidative stress induces both antioxidant reaction and autophagy, which leads to excessive production of active substances and oxidative damage to macromolecules (21).

Bone marrow mesenchymal stem cells are exposed to radiation, which will affect their survival and differentiation potential and lead to bone loss (161). Radiation can cause DNA damage on bone marrow mesenchymal stem cells, chromosome aberration, reactive oxygen species and cell senescence, which hinder the proliferation ability of bone marrow mesenchymal cells (162). In addition, radiation has a great influence on the differentiation of bone marrow mesenchymal stem cells, which will lead to the first choice of bone marrow mesenchymal stem cells to differentiate into adipocytes instead of osteoblasts, and finally lead to fat accumulation (163). Recently, some scholars have shown that BMSC-Exos can treat the effect of radiation on the differentiation of bone marrow mesenchymal stem cells. Liu et al. (164) transplantation of BMSC-Exos to save osteoporotic phenotype of recipient bone marrow mesenchymal cells improves bone through epigenetic regulation. In addition, Liu et al. (165) found that BMSC-Exos transplantation can prevent femoral head necrosis. The main preventive mechanism is to promote angiogenesis and prevent bone loss (166).

### 8.1 MSC-EVs and MSC-Exos regulate oxidative stress-related indicators in bone

It is known that 4-hydroxynonenal (HNE), a product of lipid peroxidation, can form a variety of protein complexes, which affect the activity and physiological function of OA chondrocytes. It is found that EVs treatment of OA chondrocytes can significantly form HNE complexes (160). Members of the POD (Prdx) family participate in the fight against ROS-induced cartilage injury. The exact mechanism of antioxidant protection of Prdx6 has not been clarified. Some scholars suggest that it may be directly scavenging low molecular weight peroxides and phospholipid peroxides. The protein expressed the activities of POD phospholipase A2 and lysophosphatidylcholine acyltransferase, which participated in the repair of cell membrane (167).

Mesenchymal stem cell injury is an important pathological mechanism of radiation-induced bone loss. Radiation can cause bone marrow mesenchymal stem cells to produce reactive oxygen species. Excessive ROS can lead to DNA damage (163, 168). Therefore, it is extremely important to treat radiation-induced bone loss and remove reactive oxygen species and DNA damage. Studies have shown that MSCs-Exos has excellent effects on oxidative stress and alleviating DNA damage. After irradiation, the kinds of reactive oxygen species will cause cell damage. DCF fluorescence in BM-MSCs is significant after exosomal treatment. The results of Western Blot also showed the expression of antioxidant proteins after co-incubation with exosomes. These results indicate that exudate can enhance the antioxidant capacity of BM-MSC after irradiation (166).

ATF6 is the target gene of miR-31-5P. When miR-31-5P is elevated, ATF6 does not promote endoplasmic reticulum stress of endothelial progenitor cells, which leads to apoptosis and calcification of endothelial progenitor cells. Recently, it has been reported that oxidative stress induces endoplasmic reticulum stress of endothelial progenitor cells (169). MSC-Exos down-regulates the expression of ATF6,CHOP,XBP1 and GRP78, suggesting that MSC-Exos has protective effect on endothelial endoplasmic reticulum stress induced by oxidative stress (170).

In the H_2_O_2_-induced NP cell injury model, Western blot showed that the levels of apoptotic proteins such as caspase-9 and caspase-3 were significant after H_2_O_2_ treatment, however, these were inhibited by exosome pretreatment and positive cells stained by TUNEL Significantly (17). Daisuke Sakai et al. used a microscope to observe the status of NP and annulus fibrosus, and a histological grading system to evaluate disc degeneration (171), and found slight changes in NP organization after exosome treatment. Using both X-ray and MRI examinations, exosome-treated disc height decreased more slowly at 2, 4, and 8 weeks, and IVDD treated with exosomes showed significantly higher intensity, suggesting that exosomes can delay Progression of IVDD (17).

### 8.2 MSC-EVs and MSC-Exos in the treatment of bone inflammation induced by oxidative stress

A great deal of evidence shows that inflammatory mediators can induce oxidative stress, which leads to the decrease of chondrocyte viability and the change of chondrocyte function (160). Proinflammatory cytokines produced by different joint cells can promote cartilage degradation. IL-1β stimulates OA chondrocytes to produce inflammatory cytokines. MSCs-EVs, which can exert anti-inflammatory and anti-catabolic effects. IL-6 and different cytokines can induce collagenase to produce cartilage degradation (160) and inhibit the expression of type II collagen (172). EVs can release IL-6. MSCs-EVs also demonstrated the release of MMP-13, a major collagenase that degrades type II collagen and promotes the development of OA chondrocytes into a state of like differentiation (173). Therefore, EVs can not only produce inflammatory mediators, but also control the consequences of mediator activation of cells.

NLRP3/IL-1 β plays a key role in inflammation through TXNIP activation (174). In H_2_O_2_-treated NP cells, H_2_O_2_ was significantly associated with inflammatory activation of the genes IL-1 β,TXNIP and NLRP3, which were inhibited by exosomal preconditioning. Western Blot and cellular immunity also showed the same results indicating that exosomes attenuated H_2_O_2_-induced activation of TXNIP-NLRP3 inflammatory corpuscles (17).

### 8.3 MSC-EVs and MSC-Exos down-regulate bone oxidative stress injury through mitochondria

Exosomes have obvious protective effect on the deterioration of mitochondria, exosomes can down-regulate ROS level and decrease apoptosis of NP cells, which can be seen from the low expression of caspase-3 and caspase-9. In addition, exosomes successfully expressed NLRP3 and TXNIP, thus inhibiting the decomposition of IL-1 β (17). Although systemic delivery of exosomes is generally considered to be the simplest, biological distribution indicates accumulation in the liver, spleen and lungs (175). Particularly when the avascular nature of IVDD is considered, local delivery of the subplate region is considered to be a good alternative. Previous evidence *in vivo* and *in vitro* indicates that oxidation products are widely present in IVDD (176). Cardiovascular calcification induced by oxidative stress products (177) and apoptosis and calcification of endothelial progenitor cells. These studies suggest that oxidative stress is a common pathological condition for apoptosis and calcification, including endothelial progenitor cells (178).

TEM showed that exosomal treatment alleviated mitochondrial morphological abnormalities and mitochondrial cristae breakage and disappearance induced by H_2_O_2_. Exosomal pretreatment also inhibited the production of mitochondrial ROS in NP cells induced by H_2_O_2_ (17). Proteomics MSC-Exos showed that 10.7% of exosomal proteins originated from mitochondria and 14.3% of exosomal proteins participated in ATP binding. In addition, 3.8% of exosomal proteins were involved in metabolism. It is important that MSC-Exos are enriched in different parts of mitochondria, including mitochondrial inner membrane, envelope, matrix, outer membrane and nucleoids. All in all, these data suggest that MSC-Exos may provide NP cells with mitochondrial proteins. Damaged mitochondria can be recovered by this treatment (17).

The progression of degenerative NP cells is accompanied by matrix degeneration caused by inflammation (179, 180). In order to study whether MSC-Exos can enhance mitochondrial biogenesis, Pengfei Chen et al. discovered that 10.3% of exosomes were derived from mitochondria through proteomics. Molecular function showed that 15.4% of exosomal proteins were involved in ATP binding. The enrichment of GO pathway also showed that mitochondrial fragments including mitochondrial inner membrane, envelope, matrix, space outer membrane and nucleoid were enriched in MSC-Exos (181).

Mitochondrial injury induced by mitochondrial electron transport chain complex I inhibitor reveals the chondroprotective mechanism of exosomes and whether it is inhibited by mitochondrial function. The exosomes restored the normal appearance of mitochondria in the chondrocytes (182). Chondrocytes treated with exosomes also showed mitochondrial and mtDNA content. In addition, rotenone treatment significantly inhibited the production of mitochondrial ROS in chondrocytes, which was significantly inhibited by exon treatment. The intracellular ATP level of exosomes-treated chondrocytes was 21% higher than that of rotenone-treated chondrocytes in function. These results indicate that MSC-Exos provide mitochondrial proteins to chondrocytes and thus restore damaged mitochondria (181).

The targeting and homing ability of exosomes is particularly important for their treatment in the locomotor system. Exosome hydrogel scaffolds are able to penetrate deeper into the wound and connect to the injury site when treating injuries in the locomotor system, and exosomes are homed to the peri-wound area for a more effective therapeutic effect. The combination of exosomes and materials and translation to clinical applications may be a hotspot for future research (183).

## 9 Senescence

Aging has been a topic of great concern to human beings. Aging is accompanied by the accumulation of aging cells, which changes the communication between cells and damages the homeostasis of tissues and the regeneration potential of organs (184). Recently, MSC-EVs have proved to be more effective and challenging than current stem cell-based treatments. Extracellular vesicles contain cell-specific proteins, lipids and nucleic acids, which may be released and absorbed by all types of cells to induce functional changes through horizontal transfer of their cargo. Non-aging mesenchymal stem cells cultured in low physiological oxygen tension (3%) to premature aging mesenchymal stem cells. Extracellular vesicles cultured in high oxygen (the usual oxygen culture condition is 21%) have many beneficial characteristics (185). Free radical theory holds that oxidative stress induced cell damage is one of the main causes of various senile diseases, which changes the biological structure and function (186). In particular, the cells treated with H_2_O_2_ will produce a large amount of reactive oxygen species (ROS), and the excessive accumulation of ROS will damage the macromolecular function and membrane system of cells, which will irreversibly damage various senile diseases and senescence (187, 188).

### 9.1 MSC-EVs and MSC-Exos inhibit senescence through ROS

Aging induces many cell disorders. The regeneration of mesenchymal stem cells in old age is a hot spot in regenerative medicine research in recent years. MSC-EVs have become a new tool for stem cell regeneration because of their systemic effect and safe gene transfer ability (189). In the present study, the role of aging-related ROS in the function and regeneration of mesenchymal stem cells in infant EVs was studied. The data clearly showed that the elderly MSCs showed down-regulation of SOD1 and SOD3, which led to ROS elevation and down-regulation of MEK/ERK pathway, which was related to the impaired ability of MSCs to necrotic area in flap model. In addition, edaravone or co-overexpression of SOD1 and SOD3 can save ROS increase and cell senescence of mesenchymal stem cells in old age, thus improving their function (190). It is worth noting that EVs derived from infant bone marrow mesenchymal stem cells in type 1 and type 2 diabetic mice can revitalize elderly bone marrow mesenchymal stem cells by inhibiting ROS production and accelerating cell aging to promote proliferation and *in vivo* function (191).

EVs are a powerful tool that not only inhibits ROS production, but also restores altered intercellular communication, improves stem cell function and stem cell quality, and thus delays stem cell failure in aging. It has been shown that treatment of senescent MSCs with non-senescent MSC-EVs can induce glycolytic oxidative phosphorylation of SA-β-galactosidase activity and over-expression of pluripotent factors (OCT4, SOX2, KLF4 and cMYC or OSKM). In addition, the cargo of these EVs induces up-regulation of miR-302b and HIF-1 α levels in target cells. It is concluded that miR-302b triggers the up-regulation of HIF-1 α and activates different pathways to delay premature senescence, improve stem and transform energy metabolism into glycolysis (192).

### 9.2 MSC-EVs and MSC-Exos cell proliferation inhibits senescence

miR-302b can proliferate cells and protect cells from oxidant-induced death of human mesenchymal stem cells (193). Kim, J. Y et al. studied ROS levels and cell death of human dental pulp stem cells (hDPSCs) after EVs treatment. It was found that the cells cultured under physiological hypoxia showed ROS and apoptosis levels compared with those cultured under 21% oxygen. Interestingly, no changes in ROS levels were observed after treatment, and cell cycles parallel to these observations showed no difference in G0/G1 phase, S phase and G2/M phase after EVs treatment. Cells cultured at 3% O_2_ showed higher levels in G0/G1 phase, S phase and G2/M phase, which indicated the overall proliferation of cells (191). Studies have shown that EVs can promote the function of aged AT-MSCs by inducing proliferation and up-regulating the expression of damaged cytokines, thus promoting the necrotic area of aged AT-MSCs in type 1 and type 2 diabetic mice. EVs significantly increased the proliferation of AT-MSCs in the elderly and increased the number of β-gal positive AT-MSCs in the elderly. Due to the up-regulation of SOD1 and SOD3 protein expression, the accumulation of ROS in aged AT-MSCs cells was induced by the addition of EVs. In addition, the addition of EVs up-regulated the expression of wound healing related cytokines (SDF-1VEGFAng1 and Flk1) in the elderly AT-MSCs. Transplantation studies show that the ability of aged AT-MSCs to show obvious necrotic area of flap mice after adding EVs is similar to that of infant AT-MSCs (191). Impaired expression of growth factors responsible for homing (SDF1) and angiogenesis (VEGF, Ang1, bFGF) was observed in elderly AT-MSCs. These growth factors are involved in regulating the function of EC and endothelial precursor cells (EPC) (191).

## 10 Immune

**Figure 2:**
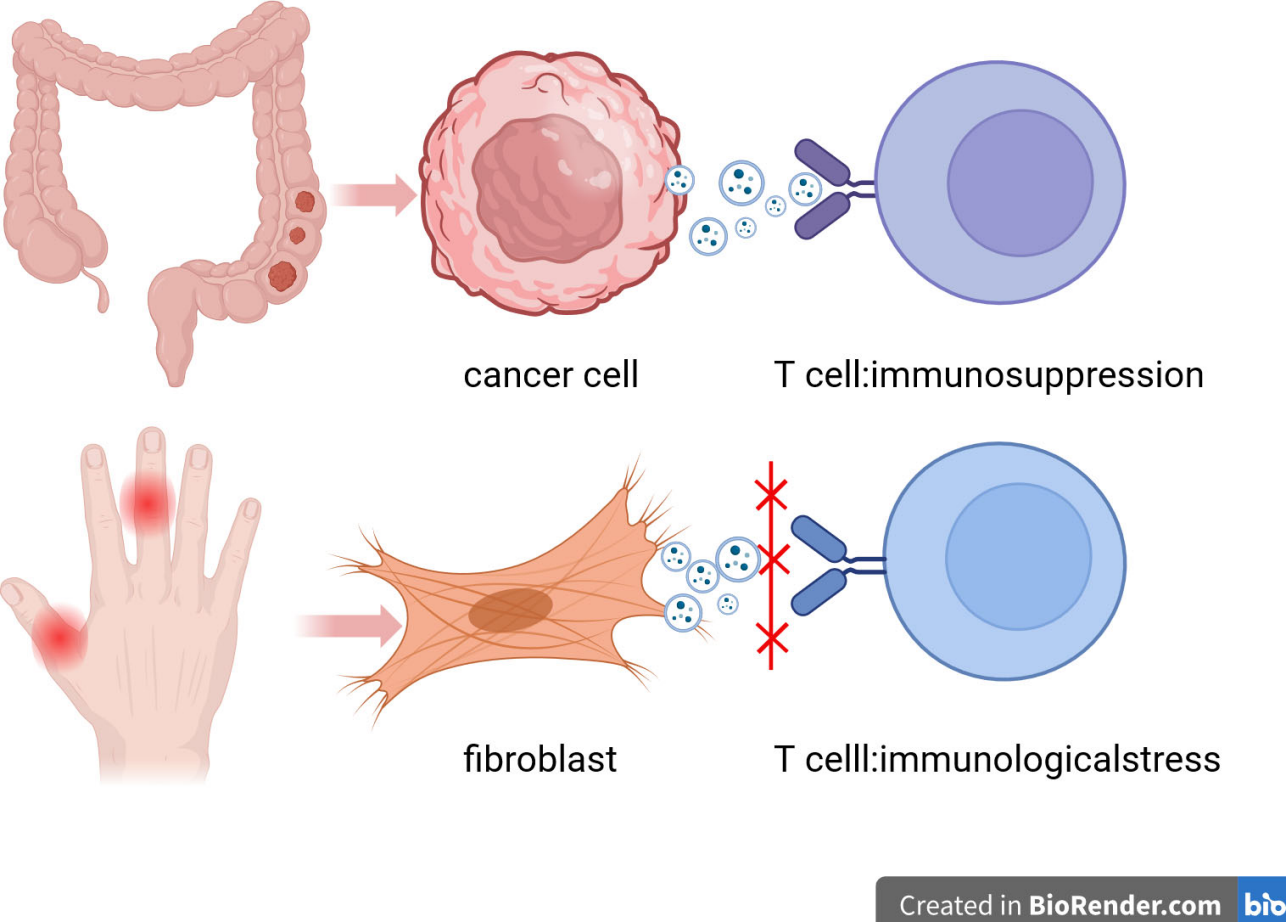
Immunosuppression and immune activation of exosomes. This figure describes the immunosuppressive effect of exosomes in cancer and the activation of immune response in synovial fibroblasts of patients with rheumatoid arthritis. Exosomes can help tumor cells escape killing by using human immunosuppressive mechanism. Exosomes can also be used as blockers of immune checkpoints to activate the immune response of T cells to kill tumor cells .

Studies have shown that MSC-EVs and MSC-Exos play both immune activation and immunosuppressive functions in cancer. The immune activation of exosomes mainly depends on the antigen presentation of exosomes, and the immunosuppression of exosomes mainly depends on the ligand protein and miRNA carried by exosomes (194). It has been reported that the existence of exosomes can provide several different mediators for cancer cells to form tumorigenic microorganisms, which belongs to the immunosuppressive effect of exosomes in cancer (195, 196). Many scholars have taken this as the breakthrough point to study cancer treatment. For example, Giovanna Andreola reported that FasL-positive exosomes released by melanoma cells can induce apoptosis of FasL-mediated Jurkat T lymphocytes (197). Phenotypically similar pro-apoptotic exosomes in the plasma of cancer patients indicate that these exosomes have a potential role in regulating host immunity and that they may become prognostic markers (198). Exosomes not only play an immunosuppressive role, but also play an immune activation role through other mechanisms. For example, exosomes released by mycobacterium-infected macrophages contain components that promote activation of adjacent uninfected macrophages (199) and many other exosomes-mediated to promote immune responses during infection with different types of microorganisms (200). In addition, synovial fibroblasts from patients with rheumatoid arthritis have been shown to release exosomes containing membrane-bound TNF-alpha that inhibit activation-induced cell death in CD4 T cells (201). Many of these studies suggest that exosomes may operate simultaneously in congenital and adaptive immune activation (202, 203) (**Figure 2**).

## 11 Conclusions and future directions

In conclusion, MSC-EVs and MSC-Exos are closely related to the regulation of oxidative stress injury in many systemic diseases. It has been fully verified in both cell model (**Table 1**) and animal model (**Table 2**). Oxidative stress can cause inflammatory factors to damage mitochondrial function, inhibit cell proliferation and enhance cell apoptosis. Inhibition of oxidative stress injury is of great significance to the treatment of various systemic diseases (233).

**Table 1:** Cell model: Summary of the mechanism of MSC-EVs and MSC-Exos in the treatment of oxidative stress-related diseases.

Disease	Disease Model	Source of MSC	Excreta	Target Cell	Oxidative stress mechanism	Therapeutic Effect	Reference
liver injury	APAP and HP injury-induced liver cells	BM(rat)	Exos	HepG2	ROS↓	Necrosis and apoptosis↓ Cell viability↑LDH activity↓	(204)
ALI	Erastin-induced ferroptosis	BM(mice)	Exos	Hepatocyte	Nrf2↑Keap1↓ROS↓ GSH↑MDA↓	GPX4↓SLC7A11↓5-LOX↑Fe^2+^↓ Cell death↓	(205)
liver fibrosis	CCl4 induced liver fibrosis in mice	HUC(human)	Exos	human HSCs line LX-2	BECN1↑xCT↓,GPX4↓ROS↑ p-MLKL↑LC3B↑	Cell death↑α-SMA↓	(77)
liver injury	CCl4/H_2_O_2_-Induced liver cell	HUC(human)	Exos	L02 Liver cells	ROS↓MDA↓GPX1↑ Mitochondrial membrane potential↓GST↓	Cell viability↑Cell apoptosis↓ p-ERK1/2↑bcl-2↑p-IKKB↓p-NFkB↓ Casp-9↓Casp-3↓	(70)
Ischemic cerebrovascular disease	tMCAO mouse	BM(mice)	Exos	H/R-injured ECs	miR-132-3p↑RASA1↓ RAS↑P-PIK3↑ p-Akt/Akt↓ROS↓ peNOS/eNOS↓	Apoptosis↓ Paracellular permeability↓ ZO-1↑ Claudin-5↑	(206)
Myocardial i8509schaemia reperfusion	H_2_O_2_-induced MIRI	BM(rat)	Exos	H9C2(rat)card-iomyocytes	ROS↓	Cell viability↑apoptosis ↓ Beclin-1↑LC3B-II↑p62↓ Autophagosomes↑ Autolysosomes↑ p-mTOR/mTOR↓p-Akt/Akt↓ p-AMPK/AMPK↑	(48)
COVID-19	LPS-induced A549 cells and PBMC	HPP	Evs	Alveolar basal epithelial cell	/	IL-8↓	(28)
ALI	LPS-induced ALI	UCB(human)	Exos	NR8383 cell	SOD↑GSH↑ MDA↓	miR-22-3p↑FZD6↓TNF-α↓IL-1β↓ IL-6↓Proliferation activity↑ Apoptosis↓	(32)
Neonatal hyperoxic lung injuries	H_2_O_2_-induced lung injury	UCB(human)	Evs	Rat lung epithelial cell line L2	/	VEGF↑ Cell survival↑	(43)
Radiation-induced lung injury	Radiation-induced injury	HP(human)	Evs	HUVECs	DNA injury↓ γ-H2AX↓	Senescent cells↓ATM↓P53↓P21↓ Inflammation-and fibrosis-related genes↓ Senescent fibroblast cells↓miR-214-3p↑	(207)
ALI	LPS-induced lung injury	UCB(human)	Evs	RAW 264.7	Nrf2↓HO-1↓ HMGB1↓8-OHDG↓	Promoted the polarization of macrophages from the M1 to the M2 phenotype CD86↓Arg1↑TLR4↓TNFα↓IL-1β↓	(208)
OA	IL-1β-induced oxidative stress	AD(human)	Evs	Chondrocytes	Oxidative stress↓ prdx6↑	LC3B↑IL-6↓MMP-13↓atg5↑P62↑ The number of associated autophagosomes↑	(160)
IDD	H_2_O_2_-induced oxidative stress	VB (human)	Exos	EPCs	/	Runx2↓caspase-3↓caspase-7↓ caspase-9↓miR-31-5p↑ATF6↓ ER-stress-related apoptosis and calcification↓	(170)
IDD	H_2_O_2_-induced inflammation	C57BL/6 mice	Exos	NP cell	Mitochondrial ROS production↓	caspase-9↓caspase-3↓iNOS↓IL-6↓ MMP3↓MMP13↓SOX9↑Col2a1↑ IL-1β↓TXNIP,NLRP3↓	(17)
Radiation bone loss	Irradiation-induced injury	BM (rat)	Exos	BM-MSC	ROS↓ SOD1↑ SOD2↑CAT↑	γ-H2AX↑Cell proliferation↑ Rb↓p53↓p21↓p16↓PPARγ↓ Ebf1↓ RUNX2↑ OPG↑Calcium deposition↑	(166)
Degeneration of cartilage	IL-1β-stimulated chondrocyte model	BM (rat)	Exos	Chondrocyte	Mitochondrial ROS↓	MMP13↓ADAMTS-5↓ COL2A1↑ATP↑ Restored mitochondria exhibiting a normal appearance Increased mitochondrial mass and mtDNA content	(181)
Epilepsy	H_2_O_2_-induced injury	UCB (Human)	Evs	Hippocampal neurons	FRAP↑CAT↑SOD↑ GSH-PX↑ROS↓DT↓ 8-OHdG↓4-HNE↓	SAMPs↓iNOS↓HMGB1↓HO-1↓Nrf2↓ Late apoptosis↓Nuclear translocation↓ Amplitude (ΔF/F) ↑MMP↑ TOM20↓FIS1↓COX IV↓	(112)
Neurological diseases	LPS-stimulated hippocampal astrocytes	UCB (Human)	Exos	Hippocampal astrocytes	Nrf2,Keap1,HO-1↓	Cell viability↑GFAP↑C3↓CD81↓ki67↓ TNFα↓IL-1β↓MMP↑p-P65/P-65↓ NF-κB↓GFAP↓ Nuclear translocation of Nrf2 and P-65 ↓	(127)
Diabetic ulcers	HDF/H_2_O_2_+HDF-induced Glucose (150mM) hyperglycemic	HF (Human)	Evs	HDF	/	The survival rate of AT-MSC and HF-MSC treated HDF cells under high glucose condition was higher than that of the positive control group.	(144)
Oxidative stress	H_2_O_2_-induced NHDFs	AD (Human)	Exos	NHDFs	Cell viability↑	Aquaporin 1↑Aquaporin 3↑ Hyaluronic acid↑SIRT1↑	(150)
Senescence	H_2_O_2_/HG-induced HUVECs	HUC,AD,BM (Human)	Evs	HUVECs	Mitochondrial ROS↓	SASP↓IL-1α↓IL-6↓IL-8↓ Aging HUVECs↑ miR-146a↑OCR↑	(148)
Photoaging	UVB radiation-induced dermal fibroblast photoaging	HUC,DF (Human)	Evs	HDFs	ROS↓ GPX-1↑	MMP-1↓,Col-1↑ SA-β-gal-positive cells↓ EVs increased cell proliferation and prevented UVB-induced cell cycle arrest	(139)
Fibrosis of the Kidney	Enicillin+streptomycin/CO_2_-induced Human renal proximal tubule epithelial cells	HP (Human)	Evs	HK-2	OXPHOS↑ ATPB↑SDHB↑COX IV↑ ROS↓	Restored morphological alterations of mitochondrial damage.	(209)
Oxidative stress	H_2_O_2_-induced keratinocytes	HUC (human)	Exos	Keratinocytes	DCF↓8-OHdG↓ FRAP↑GSH-PX↑SOD↑	GLUT1↓Calcium influx↑MMP↑ NRF2↓KEAP1↓HO-1↓NQO1↓	(23)
Chronic granulomatous disease	Chronic granulomatous disease patients’ neutrophils of their heparinized blood	AD (human)	Exos,Evs	Neutrophils in heparinized blood	CGD↑ SOD↑	Average number of yeasts that neutrophils ingested↑NBT↓	(210)
Pregnancy inflammation	LPS/AF-MSC-induced inflammatory trophoblast cells	AF (human)	Exos	HTR8/SVneo HTR8/Svneo	Inhibition of miR-548e-5p induced oxidative stress and reduced MMP in HTR8/SVneo cells.	NF-κB↓TRAF6↓IRAK1↓IL1β↓IL6↓IL8↑ miR-146a-5p↑miR-146a-3p↑ miR-146b-5p↑miR-548e-5p↑ AKT↓JNK↓ERK1/2↓P38↓	(211)
AKI	H_2_O_2_-induced HK-2	BMSC(mice\rat\ human)	Evs	HK-2	/	TFAM/TOM20protein,mtDNA↑Mitochondrial integrity in injured HK-2 cells↑ATP production respiration rate↑	(89)
Ischemia and reperfusion	Primary hippocampal cells and OGD/R	BMSC (rat)	Exos	Hippocampal cells	ROS↓ SOD↓	Nrf2↓GPx↓DJ1↑OP A1↑Mfn1↑Mfn-2↑ LRRK2,PINK↓	(212)
AD	AβOs-reduced rat hippocampal neurons	BMSC (rat)	Evs	Hippocampal neurons	H_2_O_2_↓O_2_↓ROS↓	MSCs blocked the reduction in PSD-95 levels and loss of synapses induced by AβOs	(213)
Injury of isolated hearts after cold storage ex vivo	The H9c2 rat cardiomyoblast cell line subjected to cold storage	BMSC(human),AD (human)	Exos	Cardiomyoblast cell	ROS production↓	Circadian pathways↑Mitochondrial activity↑Per2↑ Left ventricular function↓Apoptosis↓MtMP was preserved.	(214)
AMI	H/R induced human cardiomyocyte AC16 cell line	BM (human)	Exos	Heart muscle cell	LDH,MDA↓ SOD↑	Bcl-2↑ Baxc-caspase 3↓ Lnc A2M-AS1↑	(54)
Acute Myocardial Infarction(AMI)	Oxygen/Glucose Deprivation (OGD) Procedure-subjected NRCM	DP (human)	Evs	NRCM	ROS,LDH↓	Apoptosis↓ α-SMA↓Collagen protein↓	(55)
AIC	DOX/specific-induced pluripotent stem cell–derived iCMs	BM(human)	Evs	iCMs	ROS↓	contractility↑ATP↑Mitochondrial biogenesis↑Cardiomyocyte viability↑iCM viability↑Attenuated apoptosisapoptosis was inhibited by MSC-EV.	(215)
MI	Notch1 gene-deleted/N1ICD-over expressed C-MSCs	CMSC(mice)	Evs	CMVECs HAECs	/	The apoptosis of endothelial cells↓CM↓	(64)
Aging	Elderly AT-MSCs	AT	Evs	Aging AT-MSCs	ROS↑	ROS↑IL6↑IL8↑CCL5↑CCL3↑ SDF1↓VEGF↓Ang1↓bFGF↓OCT4↓	(191)
Aging	Elderly MSCs	DP	Evs	Aging MSC	OXPHOS↓	SA-β-galactosidase↓ SOX2↓KLF4↓cMYC↓ OSKM↓miR-302b↑HIF-1α↑Glycolysis↑	(192)
Urinary stone	Oxalate and COM crystals-induced HK-2 cells	HUC (human)	Evs	HK-2 cell	LDH↓H2O2↓ MDA↓ROS↓	HK-2 cells viability↑ Cytoplasmic and nuclear N-cadherin↓ ZO-1↑	(216)
SS	Myeloid-derived suppressor cells	OE	Exos	Suppressor cells	ROS↑CD40↓CD80↓ CD86↓MHCII↓	Proliferation of MDSCs↑CD4+T↓ Arginase activity↑NO↑	(217)
Hypoxia-Reperfusion injury	H/R-induced cardiomyoblasts (H9c2)	BM(rat)	Exos	H9c2 cells	miR149-5p↑let-7c-5p↑	β-catenin↑ Faslg↓	(218)
Ischemia/reperfusion injury	Lung I/R model and *in vitro* H/R	BM(mice)	Exos	Primary Lung Microvascular Endothelial Cells	PTEN↓PDCD4↓	Lung wet/dry weight ratio↓ M1 polarization↓ M2 polarization↑	(37)
Diabetic retinopathy	STZ-established diabetic retinopathy	HUC (human)	Evs	.	ROS↓MDA↑SOD↑ NRF2↓GPX1↑NQO1↑ HO-1↑	/	(219)
Heart Failure	oxygen–glucose deprivation (OGD)-induced damages to HL-1 cells	BM(mice)	MV,EV,CM	HL-1 cells	SOD↑GSH-PX↑Bcl2↑ IκBα↓p65↓LDH↓MDA↓ SOD↑GSH-PX↑Bax↓ Cleaved caspase-3↓Bcl2↑ GSHPX↑	/	(220)
Neutrophil function and apoptosis	human adipose tissue MSCs-isolated Exosomes and CM	AD (human)	Exos and CM	Neutrophil	ROS↑ Apoptosis of neutrophils↓	Phagocytosis percentage↑ Phagocytosis index↑	(221)
PD	6-OHDA-induced PD cell	HUC (human)	Evs	Neuroblastoma cell line SH-SY5Y	SOD↑MDA↓ ROS↓miR-181a-2-3p↑	EGR1↓NOX4↑ SH-SY5Y cells↑Apoptosis↓	(222)
SCI	LPS-induced differentiated PC12 cells	BM (rat)	Exos	PC12 cells	EXO-TCTN2↑ SOD↑MDA↓ miR-329-3p↑	IL-6↓TNF-α↓Bax↓Bcl-2↑ MEG3-WT↓WT-IGF1R 3’ UTR↓	(223)
Hippocampal damage due to diabetes	STZ-induced Hyperglycemia	BMSC (rat)	Exos	Primary rat astrocytes	TNF-α expression↓ miR-146a↑IRAK1↑ TRAF6↑ NF-κB expression↓	Diabetic wound healing ↑	(224)
MI	Trypsin and collagenase II-induced MI	BM (human)	Exos	NRCMs	MIF↑LVEF↑LVFS↑ The level of ROS↓	Apoptosis of NRCMs ↓ The scar size in MI↓Infarct size↓	(225)

BM (Bone marrow), HUC (human umbilical cord), OE (Olfactory ecto), DP (dental pulp), HPP (Human postpartum placentas), UCB (human umbilical cord blood), AF (amniotic fluid), AD (adipose tissue), VB (vertebral body), HP (Human placenta), DF (dermal fibroblast), HF (hair follicle), Acetaminophen (APAP), hydrogen peroxide (HP), Acute liver injury (ALI), Glutathione-S-transferase(GST), Hypoxia/reoxygenation (H/R), Transient middle cerebral artery occlusion(tMCAO), Myocardial ischemia reperfusion injury (MIRI), Peripheral blood mononuclear cells (PBMC), Line of rat pulmonary macrophages(NR8383)cell, Osteoarthritis (OA), Intervertebral Disc Degeneration(IDD), Endplate chondrocytes (EPCs), Nucleus pulposus (NP), Mitochondrial O2 consumption rate(OCR), Acute Kidney Injury(AKI), Oxygen-glucose deprivation/reperfusion (OGD/R), Alzheimer’s disease(AD), Amyloid-β-peptide(AβOs), mitochondrial membrane potential (MtMP), Acute Myocardial Infarction(AMI), Neonatal rat cardiomyocytes(NRCM), Cardiomyopathy(AIC), Cardiomyocytes(iCMs), Myocardial infarction(MI), Human aortic endothelial cells (HAECs), Mouse Aortic Endothelial Cell (CMVECs), Sjogren’s syndrome (SS), Streptozotocin(STZ), Parkinson’s disease (PD), 6-hydroxydopamine (6-OHDA), Traumatic spinal cord injury (SCI), Neonatal mice cardiomyocytes (NRCMs). ↓, decline; ↑, increase. /, this mechanism is not included.

**Table 2:** Animal model: Summary of the mechanism of MSC-EVs and MSC-Exos in the treatment of oxidative stress-related diseases.

Application	Model	MSC Source	Excreta	Effect of MSC treatment	Antioxidant mechanisms	Reference
PD	6-OHDA-induced PD (mouse)	In mouse brain tissue SN	Evs	miR-181a-2-3p↑EGR1↓ NOX4↓p-p38↓	Contralateral rotation↓α-syn↑4-HNE↓ Dopaminergic neurons↑TH expression levels↑	(222)
SCI	Contusive SCI (rat)	BM(rat)	Exos	TCTN2↑GFAP↓CCL2↓	LPS-stimulated NHAs viability↓	(223)
Hippocampal damage due to diabetes	diabetic STZ-induced hyperglycemia (rat)	Endogenous BM/stromal cells	Exos	Oxidative stress↓ Synaptic density↑ TNF-α expression↓	Synaptic plasticity in the CA1 region↑	(224)
MI	An acute MI model was created by ligation of the LAD coronary artery in adult (rat)	BM(human)	Exos	MSC‐exo↑ level of Mfn2↑ level of Fis1↓	Mitochondrial fragmentation↓ ROS generation↓	(225)
DM	STZ-induced DM (rat)	BM(rat)	Evs	Corticosterone↑ TSH↑	Thyroid H2O2 generation↓ NOX2 mRNA levels↓TPO activity↓ The pituitary 5′-deiodinase activity	(226)
AKI	AKI model(rat)	HUC	Evs	SOD↑Nrf2↑ARE↑	Apoptosis were mitigated TUNEL positive cells↓sNGAL levels↑	(227)
Liver injury	Partial hepatectomy and ischemic injury/Carbon tetrachloride intoxication-induced liver failure(rat)	BM(rat)	Exos	ROS↓	liver regeneration rate ↑AST↓ALT↓bilirubin↓ Albumin↑PCNA↑cell death↓8-Ohdg↓	(204)
ALI	D-GaIN/LPS-induced ALI(mouse)	BM(mouse)	Exos	ROS↓P62↑	AST↓ALT↓TNF-a↓ IL-6↓MCP-1↓MDA↓GSH↑ Liver weight/body weight ratio↓	(205)
Liver damage and iron death	CCL4-induced liver fibrosis(mouse)	HUC	Exos	BECN1↑xCT↓ GPX4↓ROS↑	α-SMA↓ Collagen deposition↓	(77)
Liver injury	CCL4-induced Acute Liver Injury (mouse)	HUC	Exos	/	Hepatocyte denaturation↓ Hepatic lobule destruction ↓ Survival rate of mice↑	(70)
Cardiac ischemia-reperfusion	I/R model was developed by left anterior descending coronary artery occlusion (mouse)	BM (mouse)	Exos	miR-182-5p↑ GSDMD↓ ROS↓	Alleviated cardiac dysfunction MI size↓IL-1β↓IL-18↓LDH activity↓ ASC↓caspase-1↓cell pyroptosis↑ arrest↑	(49)
I/R	Focal ischemic stroke induced by tMCAO(mouse)	BM(mouse)	Exos	miR-132-3p↑ ROS↓	Apoptosis↓BBB function↑ cMVD and CBF in the peri-infarct area↑	(206)
Lung I/R	180-minutes EVLP(rat)	BM(rat)	Evs	Hspa1a↑ Sod2↑	TPVR↓Pulmonary artery pressure↓ NO total metabolites↑Nos 2↑Edn1↓Ppeak↑ Cellular metabolism↑Glucose concentration↓ Lactate concentrations↓ATP↑HA↑Has1 and 2↑ CXCL2/CINC-3↑Cxcl1↑Cxcl2↑Ccl2↑Icam1↑ Ptgs2↑Il1rn↑Il10↑Irak3↑TTP↑Socs3↑Dusp1↑	(27)
Renal I/R	Bilateral renal arteries clamping induced RIRI(rat)	BM(rat)	Exos	MDA↓HIF1α↓NOX2↓ SOD↑GPX↑CAT↑HO-1↑	Creatinine↓BUN↓Apoptosis↓Caspase-3 activity↓ Bax↓PARP1↓Bcl-2↑MPO/ICAM1/IL1β/NFκB↓ IL10↑bFGF↑HGF↑SOX9↑VEGF↑	(90)
MIR	LAD-induced I/R injury model	BM (rat)	Exos	/	LC3B↑Apoptosis↓IS↓ EF↑LVFS↑	(48)
ALI	LPS-induced acute lung injury	UCB	Exos	/	miR-22-3p↑FZD6↓apoptosis↓p-NF-κB↓	(32)
ALI	Sepsis-induced ALI model(mouse)	UCB	Evs	SOD↑GPx↑CAT↑HO-1↑ Nrf2↑iNOS↓MDA↓	Rate of survival↑Total lung injury scores↓ Wet:dry ratios↓TNFa↓IL-1b↓IL-6↓IL-10↑ MPO↓Neutrophiles in BALF↓p-ERK↓p-JNK↓ p-P38↓p-p65↓IkB-α↓	(25)
Neonatal hyperoxic lung injuries	Exposed to hyperoxia(90%)for 14 days(rat)	UCB	Evs	/	Mean linear index↓Alveolar volume↓vWF↑ Apoptosis↓IL-1α↓IL-1β↓IL-6↓TNF-α↓ ED-1-positive alveolar macrophages↓	(43)
Radiation-induced lung injury	Exposed to thoracic radiation with a total dose of 15 Gy (mouse)	HP	Evs	MDA↓	P53↓P21↓β-galactosidase↓Vascular leakage↓ The number of infiltrated inflammatory cells↓ Inflammation↓TNFα↓IL-1β↓IL-6↓IL-10↑ Tissue fibrosis level↓COL1α1↓TGF-β↓α-SMA↓ MMP-9↓Anti-fibrotic genes↑TIMP-1↑TIMP-2↑ BMP-7↑miR-214-3p↑	(207)
ALI	LPS induced lung injury	UCB	Evs	Nrf2↑HO-1↑Keap1↓ Nrf2↓Keap1↑	IL-1β↓MCP-1↓IL-1α↓TNFα↓IL-12↓IL-10↑ Pathological scores↓iNOS↓Arg1↑CD86↓CD206↑ TLR4↓NF-κB p65↓	(33)
IVDD	IVDD rat model	BM (human)	Exos	/	MRI score↓CEP and NP tissues were better preserved CEP was thicker and that the structure was more intact histological score apoptosis↓calcification↓Runx2↓	(170)
IVDD	IVDD model (rabbit)	BM (mouse)	Exos	/	DHI improved, MMP13↓Col2a1↑ Histological score improved, Proteoglycan content↑	(17)
Epilepsy	Pilocarpine-induced seizures in wild-type and AAV-injected (mouse)	UCB	Evs	8-OHdG↓4-HNE↓DT↓AMPA↓ Glut1↓iNOS↓HMGB1↓Nrf2↓ HO-1↓ Nuclear translocation of Nrf2 ↓	TOM20↓FIS1↓COXIV↓ Neuronal membrane properties and excitability were improved Reconstruction of hippocampal neuronal function	(112)
Nerve disease	Pilocarpine-induced SE(mouse)	UCB	Exos	Nrf2↓HO-1↓Keap1↑	Hippocampal reactive astrogliosis↓C3↓CD81↓ki67↓ GFAP↓TNFα↓IL-1α↓IL-1β↓GFAP↓P-65↓ Learning and memory impairment were reduced	(127)
Renal fibrosis	Renal ischemia (mouse)	HP	Evs	Hypo-EVs inhibits tubular atrophy in renal fibrosis Hypo-Evs causes the levels of blood urea nitrogen↓ Hypo-EVs suppressed the protein expression levels of vimentin	Hypo-Evs causes collagen I in the fibrotic kidney tissue ↓α-SMA ↓ Hypo-Evs makes the fibrotic kidney tissue CPT1A↑ Hypo-Evs makes ATP in the fibrotic kidney tissue↑	(209)
Light aging	Knock out NRF2 for modeling (mouse)	HUC	Exos	The epidermal thickness↓ The density of the collagen fibers↑ Inhibits cell proliferation and collagen deposition	TNFα↓IL-1β↓IL-6↓CK14↓Ki67↓P53↓P21↓NRF2↓ Keap1↑HO-1↑NQO1↑MFI positive rate of NRF2↓	(23)
Inflammation of pregnancy	LPS+ICR (mouse)	AF	Exos	/	NFκB↑NFκB↓TRAF6↑TNFα↑IL1β↑IL6↑TRAF6↓ TNFα↓IL1β↓IL6↓	(211)
RAS+MetS	MetS plus surgically induced RAS (MetS+RAS) (pig)	Swine MSC	Evs	Mitochondrial matrix density↓ Total LDL cholesterol↓ Triglyceride levels↓	miR196a↑miR-132↑miR-192↓miR-320↓ATP↑	(228)
AKI	Renal I/R injury (mouse)	BM (mouse) BM (rat) BM (human)	Evs	KIM-1↓Number of apoptotic cells↓	IL-6↓IL-1β↓ICAM1TNF-α↓TFAM↑PGC-1α↑ NDUFS8↑ATP5a1TFAM expression↑ATP↑ mtDNA copy number↑	(89)
Unilateral renal vascular disease with metabolic syndrome	A pig model of unilateral renal vascular disease with metabolic syndrome	Pig autologous fatMSC-Evs	Evs	RBF↑GFR↑Cortical microvascular and peritubular capillary density↑ Apoptosis of renal cells↓ Renal tubule injury and fibrosis↓ Superoxide nion↓Isoprostaglandin↓	VEGF↑Notch-1↑DLL4↑ Oxidative stress↓	(149)
Injury of isolated heart after external refrigeration	Isolated mouse heart (mouse)	BM (human) AD	Exos	/	TNF-α↓IL-1β↓Hspa1a↓caspase-3 levels↓ I-NDUFB8↑II-SDHB↑IV-MTCO1↑ V-ATP5A↑The production of H2O2↓	(214)
AMI	LAD(rat)	DP-derived MSC	Evs	EVs+miR-4732-3p significantly restored the systolic function of AMI reduced the area of fibrous scar tissue	/	(55)
MI	LAD(mouse)	Mouse Cardiac MSC	Evs	Reduced infarct size Promote blood vessel formation Reduce cell apoptosis Stimulate CM proliferation	/	(64)
Liver Injury	Liver tumor (mouse)	HUC	Exos	SOX9↓BAX↓bcl↑	8-OHdG↓aspase 3↓MDA↓TGFβ↓	(73)
Hepatic I/R Injury	Hepatic I/R Injury (mouse)	BM (mouse)	Evs	Caspase 3–positive cells↓ Apoptotic cells↓TNF↓IL1a↓ IL1b↓IL6↓IL12↓IFN↓ Chemoattractant protein 1↑ F4/80positive cells↑ALT↑	NF-jB↓ROS↓NLRP12↑CCL7↓NLRP3↑ Mitogen-activated protein kinase 13↑ CXCL1↑IFNb1↓IFNc↓l 1b↓IL33↓kappa B↓ IL6I↓L1b↓PTGS2↑	(72)
DR	Diabetic model (rat)	HUC	Evs	PETN↓AKT↑NRF2↑ Retinal thick-ness↑ Caspase-3 positive cells↓	NEDD4↓PCNA↑Bcl-2↑Bax↓MDA↓SOD↑NRF2↑ GPX1↑NQO1↑HO-1↑GCLC↑GCLM↑	(219)
Cerebral infarction	MCAO for cerebral infarction model (mouse)	HUC	Exos	Recover cell viability↓ Apoptotic cell numbers↓ ROS↓TLR4↓infarcted area↓ Brain water content↓ Neurological grading scores↓ FJC-positive cell numbers↓	Tnf-α and MCP-1 inhibition increased TNFα↓IL 6↓	(229)
Heart failure	Heart failure (mouse)	BM (mouse)	Exos	CD31↑CD206↑CTGF↓Bax↓ Cleaved caspase-3↑Bax↑ BCL↓IκBα↓p65↓caspase-3↓ Activity of MDA and LDH↓	/	(220)
Hepatic I/R injury	hepatic I/R injury (mouse)	human-induced pluripotent stem cell	Exos	TNF-α↓IL-6↓HMGB1↓ Caspase-3↓bax↓ALT↓ AST↓bcl↑Ki67-positive cells ↑	GSH↑GSH- px↑SOD↑MDA↓	(74)
Osteoarthritis	Osteoarthritis (mouse)	BM	Exos	MMP-13↓SOD↑NO↓MDA↓iNOS↓ COX2↓IL-1↓IL-6↓TNF-α↓	SDC1↓CRP↓	(230)
MI	model of myocardial infarction (rat)	HUC	Evs	DEF↑ FS↑ DFS↑ LVIDs↑LVEF↑	/	(231)
ED	model of internal iliac artery injury‐induced ED (rat)	BM(rat)	Exos	ICP/MAP↑CD31↑VEGFA↑ iNOS↓SOD↑8-OHdG↓	/	(232)

Parkinson’s disease (PD), Substantia nigra (SN), Spinal cord injury (SCI), Myocardial infarction (MI), Type 1 diabetes mellitus(DM), Acute Kidney Injury(AKI), Acute liver injury (ALI), Ischemia reperfusion(I/R), Ex vivo lung perfusion (EVLP), Myocardial ischemia reperfusion(MIR), Intervertebral disc degeneration (IVDD), Renal tubular necrosis rate Renal Damage Molecular 1(KIM-1), Diabetic retinopathy (DR), Erectile dysfunction (ED), Alzheimer’s disease (AD). ↓, decline; ↑, increase. /, this mechanism is not included.

In the study of the mechanism of stem cell exosomes, iron death, copper death, autophagy and proteomics may be the hot spots in the future. In the application research of stem cell exosomes, hydrogel, collagen and other materials combined with exosomes to form composite scaffolds have been carried out by many scholars in cell experiments and animal experiments, but its clinical transformation has not yet been realized. Verifying its human safety and ethical rationality is the direction that needs to be worked hard now.

Regulatory proteins and miRNA in MSC-EVs and MSC-Exos are the core of treatment. However, the same protein and miRNA seem to show different results in different studies (234). At present, no research has shown the reason for this result. Potential effects of stem cell origin, such as the age of stem cell donors, may be related to the production of oxidative stress and inflammatory mediators, which may affect their immunomodulatory function (235). It may also be caused by sex, for example, female MSCs cause greater immunomodulatory effect than male MSCs (236). For example, some studies have not clarified the effective proteins in MSC-EVs and MSC-Exos. Although researchers have used proteomic databases to show which antioxidant proteins MSC-EVs and MSC-Exos contain, further work is needed to isolate and accurately identify these proteins (237). In addition, only one dose was used in some studies, and it is necessary to explore the possible dose-dependent protective effects of MSC-EVs and MSC-Exos (17).

At present, many functions of MSC-EVs and MSC-Exos have been discovered one after another, but these studies also have some limitations, MSC-EVs and MSC-Exos may have different inhibitory effects in different cell species and animal species (16). In addition, the differences in the isolation and application steps of EVs in different studies may lead to different effects of the same regulatory protein and miRNA. In order to ensure the repeatability of the effects of MSC-EVs and MSC-Exos, it is necessary to explore and formulate a protocol to control all the steps of their isolation and application (160). Therefore, MSC-EVs and MSC-Exos in the treatment of oxidative stress injury of various systems need to use models closer to human pathology for better clinical use (235).

## Author contributions

All authors contributed to the conception and design of the review central idea. WZ wrote the first draft of the manuscript; YX and TW prepared the tables and retrieved literature; XP and YZ corrected the drafts of the paper. All authors contributed to the article and approved the submitted version.
